# Lessons learned from interdisciplinary US national science foundation research traineeship-supported graduate programs

**DOI:** 10.1371/journal.pone.0343307

**Published:** 2026-02-20

**Authors:** Jyothi Kumar, Michelle R. Worosz, Shin-Han Shiu, Pamela H. Templer, Karen S. McNeal

**Affiliations:** 1 Department of Plant Biology, Michigan State University, East Lansing, Michigan, United States of America; 2 Department of Agricultural Economics and Rural Sociology, Auburn University, Auburn, Alabama, United States of America; 3 Alabama Agricultural Experiment Station, Auburn University, Auburn, Alabama, United States of America; 4 Department of Computational Mathematics, Science, and Engineering, Michigan State University, East Lansing, Michigan, United States of America; 5 Department of Biology, Boston University, Boston, Massachusetts, United States of America; 6 Department of Geosciences, Auburn University, Auburn, Alabama, United States of America; Iowa State University, UNITED STATES OF AMERICA

## Abstract

The National Science Foundation’s (NSF) Research Traineeship (NRT) Program fosters interdisciplinarity. It is designed to train scholars with the agility to move beyond their strict disciplinary boundaries. The goal of the Program is to create innovative graduate educational opportunities for broad workforce development. We assessed the professional development skills and activities documented in the annual reports of 20 NRT projects to assess which technical and transferable skills were commonly highlighted, how these skills were integrated into their educational programming, which stakeholders were targeted, and how much time was allocated to the associated activities. We found communication (43%), job readiness (42%), and team science (26%) to be the most common professional development skills provided by the 20 NRT projects. We go into greater depth about three of these NRT projects to more deeply characterize programmatic challenges and successes. Then, we highlight strategies to manage potential points of friction and to recommend approaches that could be adopted as part of other graduate professional development projects. These innovations have the potential to promote transformative change in graduate STEAM education, nationwide.

## Introduction

Leading science organizations in the United States (US), including the National Academies of Sciences, the National Research Council, and the National Science Foundation (NSF) identified a number of increasingly complex challenges facing society including climate change, land use and land cover change, biodiversity loss, energy and infrastructure, poverty and food security, and chronic disease [1]. These challenges are intertwined with an array of economic, social, and political phenomena that have implications beyond the technical aspects of science alone [2–4]. To meet these challenges, prominent scientific voices have called for training a new generation of scholars in Science, Technology, Engineering, Arts, and Mathematics (STEAM) who would have the agility to move beyond disciplinary boundaries and connect varying theories and methods in the process of creating new knowledge. To develop the skills necessary to transcend one’s discipline and to integrate the methods and knowledge of multiple fields of study, which NSF refers to as interdisciplinarity, requires a transformative approach to graduate education [2]. One strategy intended to accomplish this transformation is the NSF Research Traineeship (NRT) Program, an initiative within the Division of Graduate Education. The goal of the NRT Program is to train emerging professionals in interdisciplinary research and to prepare them to work in a variety of career settings [3]. While isolated studies of NRT projects can be found [e.g., 5–7], there are no known studies of the NRT Program at large.

To date, NSF has invested approximately $550 million USD in the NRT, which has supported more than 200 graduate projects across diverse US institutions, and served an estimated 10,000 students (5,000 Trainees). We examined 20 of these NRT projects to assess the breadth and depth of the approaches used to promote interdisciplinarity and professional skills development. We asked how these projects might serve as a model to improve upon traditional, discipline-based training. Our analysis centered on quantifying the professional skills (e.g., communication, interdisciplinarity, team science) emphasized, identifying the activities (e.g., coursework, internship, seminar) that were used to guide skill development, and assessing the amount of time allocated to the activities. In addition, we asked which stakeholder groups (e.g., graduate students, faculty, staff, partner organizations) were targeted. We also draw on our experiences as faculty leaders and project coordinators of three NRT projects–Climate Resilience at Auburn University (AU), IMPACTS at Michigan State University (MSU), and URBAN at Boston University (BU)–to provide more comprehensive and comparative insights into the implementation of interdisciplinarity and associated professional development.

## Background

Interdisciplinary research is often used as an umbrella term for several similar concepts including multi-, inter-, and trans-disciplinarity [8] and, more recently, convergent research [6]. Interdisciplinary science, however, is typically used to describe the practice of bringing people together from different disciplinary backgrounds and integrating their knowledge and methods [9]. This approach is essential when addressing the most pressing social and environmental issues [10] as interdisciplinarity offers the benefits of increased creativity, innovation, and productivity [9]. While interdisciplinary research is widely valued, graduate degree programs are predominantly structured around traditional discipline-based departments. This siloed model is often cited as a barrier to interdisciplinary collaboration [11]. Thus, novel training programs are essential to the development of scholars who have the capacity to thrive and contribute effectively in complex settings [12]. For instance, a survey of early-career climate scientists (i.e., within 3 years of PhD) identified a need for improved training to foster interdisciplinary skills, particularly communication and teamwork [13,14]. To bridge these training gaps, scholars have advocated for a cultural shift in graduate education to better align with 21st-century needs [1,15–18]. Student-centered training approaches and T-shaped training models, those with breadth and depth, have been recommended as frameworks for said change [1,17]. The ultimate goal of these learner-centered training programs is to produce students of all backgrounds who are capable not only of meeting rigorous STEAM standards but also applying interdisciplinary knowledge across the broad range of occupations required to address vexing societal needs, globally [1].

NSF awards for NRT projects [3,12] are granted with the expectation of integrating the newly developed approaches for graduate training into their respective universities such that they last beyond the scope of the five-year grant. However, previous studies [19–22] point to numerous barriers and constraints to adapting traditional graduate education to fit this relatively new model. In fact, a predecessor to the NRT, the Integrative Graduate Education and Research Traineeship (IGERT) Program, funded approximately 125 projects and impacted more than 2,900 students over a ten-year period. Several limitations emerged regarding the impact of IGERT beyond the Trainees’ specific cohorts. For instance, the projects were not mandated to include non-Trainees, thus graduate students outside of the project lacked opportunities to benefit from the training [23]. Another shortcoming of IGERT projects was that they typically lacked plans for institutionalization and activities often ceased when the funding ended [24]. These limitations suggest that long-term project sustainability requires deliberate planning and organizational support [25]. Nevertheless, an IGERT program-wide evaluation found that participants, those who received expansive educational experiences (e.g., interactions outside their home department, participation in interdisciplinary research projects), were indeed more prepared for the workforce than non-IGERT students [24]. It was also found that the participating Trainees helped to advance interdisciplinary graduate education in their university [24].

To encourage broader graduate student impacts and to advance the institutionalization of graduate program elements, NSF transitioned IGERT into NRT in 2014, but retained many of the original program elements–professional development training, high-impact interdisciplinary research, and broadening STEM participation. Several NRT projects have since reported on their accomplishments, including programmatic aspects that were both unique to NRT and those that were carried over from IGERT. For instance, a project at the University of Maryland addressed both the challenges and benefits of student-faculty, interdisciplinary collaboration, and the institutionalization of these efforts. The project leaders highlighted key activities (e.g., regular research talks, allocating shared physical space) that helped drive institutional change and encourage adoption by other graduate programs at their institution [7]. In another NRT project, interdisciplinary research was used to enhance participation in STEM at Kansas State University where project leaders found that having the opportunity to work with people who have different styles of problem-solving was one of the Trainees’ favorite aspects of the project [26,27]. In another example, an NRT project at the University of Kentucky implemented professional development courses to focus on transferable skills (e.g., communication, management, leadership, teamwork, ethics, conflict resolution, mentoring, entrepreneurship) [6]. Findings from the evaluation of this latter project indicated that the approach allowed graduate students to work with both students and faculty from other disciplines, which positively influenced their research by including different problem solving perspectives and skills [6].

In the sections below, we begin by presenting results of our analysis of professional activity training across 20 NRT projects. We then provide a detailed assessment of three of these NRT projects: Climate Resilience, IMPACTS, and URBAN. This analysis provides insights into the ways in which the projects were structured and the potential solutions for incorporating interdisciplinary training within established models of higher education.

## Results

### Analysis of 20 NRT projects

**Fig 1:**
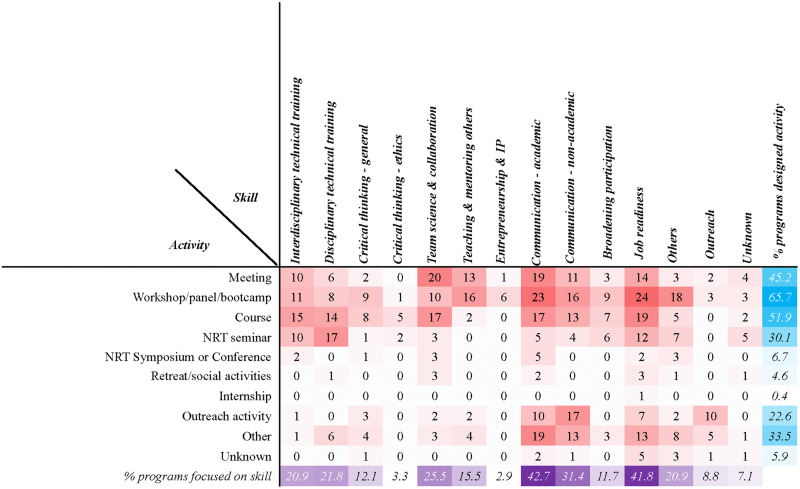
NRT project activities for training different professional skills. *Red*: shade of red indicating lower (lighter) to higher (darker) number of instances for a particular activity/skill combination. *Purple*: shade of purple indicating a lower to higher percentage of projects focused on a particular skill set. *Blue*: shade of blue indicating a lower to higher percentage of projects conducting a particular activity.

Through our survey of 20 NRT projects we found that while each project had an explicit goal of enhancing students’ capacity for interdisciplinarity, their focal skills emphasized communication to academic or non-academic audiences (43% and 31%, respectively), followed by team science and collaboration (26%), various technical skills (21%) and critical thinking (12%,), broadening participation (12%), and outreach (9%) (Fig 1). Additionally, many projects emphasized job readiness (42%). Less common was teaching and mentoring others (16%), as well as entrepreneurship (3%). A variety of miscellaneous skills were also identified including cultural competence, STEAM, and co-production of knowledge with stakeholders.

Diversity in project design was evident in the range of instructional approaches and delivery methods. In addition to meetings and seminars, the most common pathways of engagement were workshops (66%), panels (52%), boot camps (45%), and semester-long courses (30%, Fig 1). In fact, the projects dedicated most of their time to these latter activities and leveraged them to develop the majority of the targeted skills. Communication and job readiness were found to be fostered through more diverse activities compared to other categories. For example, professional competencies were emphasized through mentoring, active listening, conflict resolution, time management, networking, and job searching. Several projects also required internships (Fig 1). The data suggest that these efforts were designed for both experiential learning and the application of course material, and other discipline-based training, in order to address real-world scenarios. More than half of the projects (n = 11) engaged in activities designed for cohort building such as retreats and various social events.

**Fig 2:**
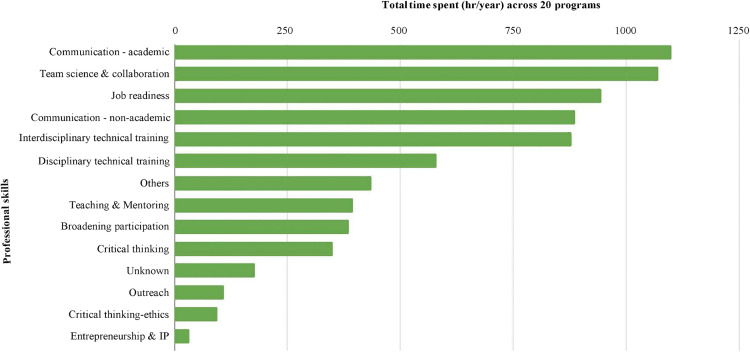
Time devoted to developing different professional skills.

Examination of time spent on tasks revealed more than half (52%) of total project effort was rooted in interdisciplinarity (i.e., communication, team science and collaboration [Fig 2]). A distant second was job readiness (12%). The time devoted to these two skill categories combined appears to be approximately 10 times more than that spent on traditional disciplinary technical training (7%). Among the interdisciplinary skill categories, most time was spent on enhancing communication to academic and non-academic audiences.

Collectively, the 20 NRT projects offered 623 separate activities across their reporting year, with an average of nearly 40 participants (Table 1). Of these activities, 65% were open to non-Trainees, including staff, faculty, and students. (Table 1). Approximately 21% engaged “other” graduate students (i.e., those who might benefit from one or more activities but are not formally enrolled in an NRT). We found that each project serves about 200 “other” students during their program per year. Another hallmark of the NRTs is the heavy involvement of faculty, project coordinators, and staff who provide Trainee support, faculty and staff professional development, and liaison with partner organizations. The data suggests that NRT projects occasionally invited university administrators, alumni, project advisory boards, and university communications specialists to also participate in their programming or events. Public stakeholders accounted for the highest participant counts per activity, which was largely driven by symposiums and outreach events.

**Table 1:** Number of Stakeholders and their Attended Activities in NRT projects.

Stakeholder category	Participants in all Activities (#)	Activities (#)	Participants per Activity (#)
Trainees	7,611	218	34.9
Other graduate students	4,090	129	31.7
Faculty	4,457	109	40.9
Project Coordinator/staff	1,242	69	18.0
Advisory Committee/evaluator	151	7	21.6
Organization offering internship	4	1	4.0
Partner organization	1,491	22	67.8
Public stakeholders	2,220	18	123.3
Others	3,250	50	65.0
Total each category	24,516	623	39.4

### Three NRT projects: Commonalities

Auburn University (AU), Michigan State University (MSU), and Boston University (BU) are Research I institutions located in the South, Midwest, and Eastern U.S., respectively. The NRT projects were Climate Resilience at AU, IMPACTS at MSU, and URBAN at BU.

Each project had an External Advisory Board, which provided support and feedback about program elements. Both IMPACTS and URBAN targeted Ph.D. students only, whereas ~30% of the Climate Resilience project Trainees were at the M.S. level. Each project was funded for five years (with a sixth-year, no-cost extension), and designed so that students could complete the required activities without delaying their progress toward graduation. While some adjustments were necessary (e.g., online on-boarding), each continued to operate during the height of the COVID-19 pandemic. Across the three NRT projects, all Trainees were required to have at least one committee member or mentor from outside their primary discipline, and all Ph.D. students participated in an internship.

Both the URBAN and Climate Resilience projects required first-year Trainees to complete an on-boarding experience during the first week of the academic year. The experiences included short presentations by project leadership that provided an overview of the mission and vision of the program, program requirements, and upcoming events and deadlines. Each project relied heavily on formal coursework for skill development, but opportunities for hands-on data collection exercises, tours of campus, team-building exercises, guest lectures, and place-based field experiences also occurred. The aim of these experiences were to deliver scientific content and to build a cohesive cohort of students for the duration of their project and beyond. Other similarities between projects included the design of a symposium or retreat as part of their professional development and creating relationships with external organizations which included outreach components for students to practice science communication skills.

Four cohorts graduated from the Climate Resilience project, and five cohorts graduated from IMPACTS and URBAN during the lifetime of the NSF grant. Funded Trainees in the Climate Resilience project were provided 2-years of stipend support, whereas URBAN and IMPACTS were provided 1-year of stipend support. Elements of each project have continued after the NSF grant ended. Project specifics are detailed in Table 2 and in the paragraphs below.

**Table 2:** Characteristics of Three NRT Projects.

		Climate Resilience	IMPACTS	URBAN
Participants (Total)	Trainees (funded and non-funded)	n = 26	n = 54	n = 40
	Faculty	n = 11	n = 29	n = 40
Disciplines		Biology; Biosystems; Crops, Soils and Environmental Sciences; Forestry; Geology; Geosciences; Public Administration; Rural Sociology; Statistics	Plant science; Computational Science	Biogeoscience; Environmental Health, Math & Statistics
Course Requirements		Science Communication (3-credits)* Natural Hazard Risk and Disaster Resilience (3-credits)* Non-disciplinary elective (3-credits) STEM Studio (2-credits)**	Foundations in Computational Plant Science (3-credits)* Frontiers in Computational Plant Science (3-credits)* Forum in Computational Plant Science (1-credit)*	Colloquium (2-credits)* Internship (1–2-credits)* Biogeoscience 4-credits) Environmental Health (4-credits) Math/Statistics (4-credits)
Meetings, Workshops, Symposiums		Meetings: lab tours, informal social activities, Workshops: Summer immersion, Structured decision-making, Metacognition, Mentoring Symposia: Annual Climate Symposium with multiple departments, external universities, an industry partner sponsor, and external stakeholders..	Meetings: focus groups with internal evaluation team members including the program coordinator; feedback from Trainees about program experiences with leadership and Trainers Workshops: Data Carpentry workshop organized by Trainees & non-Trainees Symposia: with Corteva Agriscience and another Plant Science graduate program	Meetings: Faculty/Staff Meetings each semester; Feedback sessions with faculty/staff and students each semester; Regular check-in meetings for Trainees with Project Manager Workshops: Introductory workshop, City Governance, Science Communication Symposia: Annual Symposium
Outreach and Internships		Community Science Events: 3–4 per each year Non-traditional research internship** Art Wall Development and Display	Internships with virtual and in-person in companies, organizations	Internships with government, non-governmental organizations, and private sector Internship-companion course
Institutionalized Elements		Science Communication course Annual Symposium Art wall Outreach office graduate student science communication awards Revised Ph.D. program Science Communication workshop series (micro-badge) AU Solves: University level program for co-production with community non-profits Team Science Leadership workshops	Graduate certificate in Computational Plant Science including the new courses established in the program	Courses, workshops Project Manager position

* New courses created for the respective NRT Project.

** The studio/internship was not required for M.S. level students.

**Climate Resilience Project**. The Climate Resilience NRT Project at Auburn University (2019–2025) was centered on expanding knowledge about regional climate impacts, co-production of actionable science with stakeholders, and communicating solutions to diverse audiences. As illustrated in Table 2, faculty (n = 11) and students (n = 26) were from diverse disciplinary backgrounds that were associated with numerous colleges and departments including Science and Mathematics (i.e., Geosciences, Mathematics and Statistics, Office of Diversity and Multicultural Affairs); Engineering (i.e., Biosystems); Forestry, Wildlife, and Environment; Liberal Arts (i.e., Public Administration), and Agriculture (i.e., Agricultural Economic and Rural Sociology; Crops, Soils, and Environmental Sciences). The project also received support from the Graduate School and the University’s Biggio Center for Teaching and Learning. In addition, the project established partnerships with large, regional, climate organizations and historically Black colleges and universities (HBCUs). These associations provided access to a network of experts and advisors, as well as external stakeholders.

The Climate Resilience project aimed to integrate three areas of concentration–built environment, natural systems, social systems. All Trainees were required to take three courses: Natural Hazards and Disasters, Science Communication, and an elective outside their discipline. Students at the Ph.D. level were also required to take STEM Studio, a course focused on working with a stakeholder community to examine and/or solve a problem of interest. Additionally, Trainees were required to have a thesis or dissertation committee member from outside their department and to integrate some form of interdisciplinarity into their work. Interdisciplinarity was reinforced via an extensive series (i.e., approximately 16 per year) of, often mandatory, engagements that included team-based projects.

*Skills.* Trainees and both project faculty and faculty advisors received professional development training in several areas such as mentorship, teamwork and collaboration; metacognition and structured decision-making; and co-production of knowledge. Trainees had opportunities to acquire technical competencies such as numerical modeling, geographic information systems (GIS), discourse analysis, and both science framing and messaging techniques, as well. They also engaged in substantive areas to strengthen their knowledge of key topics such as climate science, coastal hazards, and adaptation strategies.

*Activities.* Structured coursework provided the foundation for Trainees’ skill development including concise information delivery via elevator pitches and 3-minute research videos. Meetings and workshops were heavily utilized to discuss topics such as interdisciplinarity and collaboration, interact with external stakeholders (i.e., Advisory Board), and support event planning. Contribution toward the planning and implementation of an annual climate symposium was a core activity that provided Trainees with an opportunity to present their research and receive formative feedback. Another important activity was the 5-day Immersion Trainee Workshop, which served as a means of on-boarding; it provided an observational field experience related to climate impacts, risk, and recovery; it introduced students to the concept of interdisciplinarity; and it facilitated social cohesion. Trainees also collaborated on the creation and delivery of a series of outreach activities in public libraries and K-12 schools, and during science festivals (e.g., Earth Day celebration).

*Time.* Coursework comprised the backbone of the Climate Resilience project with 160 hours of formal instruction offered per year. Considerable time was also dedicated to meetings (138.5 h/yr), workshops (81 h/yr), seminars (24.5 h/yr), an annual symposium (12 h/yr), and social events (7.5 h/yr). Most activities were open to faculty, broadly, and many were made available to graduate students who were not in the project. These functions were designed to reinforce skills associated with communication to academic (269.7 h/yr) and non-academic (200.2 h/yr) audiences, disciplinary work (219 h/yr), miscellaneous research methods and analysis techniques (e.g., discourse) (123.8 h/yr), interdisciplinarity (116.2, h/yr), job readiness (113 h/yr), collaboration (94.5 h/yr), mentoring (77.5 h/yr), critical thinking (61.5 h/yr), and broadening participation (3 h/yr).

*Institutionalization.* The Science Communication course remains in place. As a substitute for the Studio, a previously existing course (i.e., Extension Methods and Programming) was modified to provide comparable skill development (e.g., outreach, non-academic communication, project planning). Another existing course (Seminar, 1-Credit) was redesigned to fill gaps in, and to extend knowledge about interdisciplinary research. Although the substantive content shifted (i.e., from climate to environment, broadly), support for an annual symposium remains and it will continue to advance interdisciplinarity. The Climate Resilience project itself was memorialized via a team-based art project. The Trainees envisioned the original content and description of a mural that was installed in a prominent campus location. Finally, expertise acquired over the course of the project was leveraged in at least two ways. At the college level, an existing interdisciplinary Ph.D. program is undergoing revision based on the lessons learned. In another case, at the University level, project faculty have informed two workshop series: Science Communication, a micro-credential and Team Science Leadership; and a community-scientist co-production project (AU-SOLVES). Each of these activities are supported by different offices at the university with limited external funds from industry, demonstrating the breadth of post-award support for institutionalization of key project elements.

*Challenges.* The Climate Resilience project saw challenges which included those related to: *COVID-19* (e.g., moving all activities online, creating team cohesion), *faculty* (e.g., finding appropriate incentives for keeping faculty engaged over the six year timespan, engaging new faculty to replace those who left the institution); *interdepartmental* (e.g., communication between departments and colleges such as securing graduate student contact information), *administrative* (e.g., structures to pay/travel reimburse Trainees who were not considered employees due to their fellowship status), *institutionalization* (e.g., finding appropriate campus offices willing to invest in post-award sustainability), and *team science* (e.g., finding meaningful projects to engage the project’s expanding disciplines).

**IMPACTS Project.** The IMPACTS project at Michigan State University (2018–2024) focused on training future scientists who can use interdisciplinary approaches to address grand challenges in plant biology and to ensure the safe, reliable, and sustainable production of food and biofuels. Participants included more than 50 Trainees and 29 faculty members. The predominant disciplines were plant and computational sciences, which originated from the colleges of Natural Science and Engineering (Table 2).

IMPACTS aimed to train Ph.D. students to employ advanced computational and data science approaches. All Trainees were required to take three core courses: Foundations in Computational Plant Science, Frontiers in Computational Plant Science, and Forum in Computational Plant Science. Alongside substantive material, these core courses included professional development topics intended to engage students from diverse disciplines and to promote interdisciplinary exchange. All Trainees were required to have a co-mentor from plant or computational science to ensure interdisciplinary expertise and guidance were incorporated throughout the completion of their respective NRT projects. Trainees were also required to participate in internships with industry and government agency partners to broaden their career perspectives.

*Skills*. IMPACTS Trainees received training that emphasized the development of interdisciplinary skills in the foundational plant science course (e.g., critical thinking, team science, collaboration, and communication with interdisciplinary audiences). In the frontiers course, emphasis emphasized team-based and project-based learning focusing on solving authentic research problems. The forum course also focused on STEM Teaching and Learning topics which led the Trainees to develop a lesson plan. Trainees through collaboration with other graduate students, Trainees organized symposia and retreats to develop organizational, leadership and management skills.

*Activities.* While coursework served as a vital foundation for the Trainees’ skill development, various retreats, symposia, and other social activities were used to reinforce their skills and overall job preparation. Project meetings and outreach initiatives, for instance, focused on communication, and as part of the Forum in Computational Plant Science course, Trainees learned how to deliver both 10–15 minute research and 3-minute lightning talks. Additional professional development activities were tailored toward Trainee career goals (e.g., interacting with experts from mid- to large- size companies). Outreach activities were also used. For instance, IMPACTS Trainees guided middle school and high school students through a paper maze to explain important concepts in coding and how to think like a programmer. This activity focused on advancing Trainees’ ability to communicate with a broader non-academic audience.

*Time.* IMPACTS project coursework consisted of 60 hours per year of formal instruction. Programming for skill development also included meetings (9 h/yr), symposia or retreats (17 h/yr), and workshops (32 h/yr). Additionally, time was allocated to develop skills in critical thinking (60 h/yr), interdisciplinary research (90 h/yr), team science and collaboration (98 h/yr), and communication for academic audiences (118 h/yr).

*Institutionalization.* Within the timeframe of the NRT project, IMPACTS was able to institutionalize the NRT curriculum and developed courses into a graduate certificate program. This graduate certificate was approved across colleges and was accessible to both Trainees and non-Trainees.

*Challenges:* One of the most significant challenges encountered during the second year of the IMPACTS project was the COVID-19 pandemic. This affected how the project was adapted and sustained throughout the pandemic. Online engagement with the Trainees declined over time, resulting in delays in project activities. For example, some of the Trainee research and internships were postponed or scaled back. A second challenge was Trainee recruitment in our second year. This issue was addressed by promoting a few IMPACTS fellowships for incoming graduate students. Finally, interaction with our advisory board was minimal, thus the project had little external input into the structure of the program.

**URBAN Project.** The URBAN project at Boston University (2017–2023) prepared students to tackle major environmental problems that confront cities by integrating tools from biogeoscience, environmental health, and statistics with external partners in government, non-governmental organizations, and the private sector. There were 40 participating Trainees and 40 faculty from the Departments of Biology, Earth, and Environment; Math and Statistics; Environmental Health; and Computing and Data Sciences during the lifetime of the NSF grant (Table 2). External partnerships were developed to aid in the implementation of interdisciplinary solutions-oriented training; Trainees completed internships with policymakers, the private sector, and non-governmental organizations. In addition to the internship, Trainees were also required to take Biogeoscience, Environmental Health, Statistics, and a Program Colloquium Course. All Trainees were required to have a dissertation member from the complementary program (e.g., a student enrolled in Biogeoscience would have a faculty member from Environmental Health).

*Skills.* Trainees received professional development training in several areas such as teamwork, collaboration, co-production of knowledge, science communication, and the workings of city governance, as well as interdisciplinarity. Students also had an opportunity to practice grant writing.

*Activities.* Structured coursework served a particularly critical role for Trainees in the URBAN project. For example, Trainees in the internship-companion course practiced elevator pitches and discussed progress in their internships. Workshops and symposia provided the foundation for Trainees’ skill development and a means of cohort building. In addition to the Introductory Workshop, there were other workshops organized throughout the year–Science Communication, City Governance, Professional Development–that included career panels with project alumni and visits to local mayoral offices, the Massachusetts Statehouse, and U.S. Congressional and federal agency offices. Small competitive grants were made available to Trainees for research and travel if they proposed interdisciplinary work and/or presented interdisciplinary work at a conference. Both students and faculty presented at an annual symposium, which provided a synthetic way to share the key elements of the project with both participants and others including members of the university’s upper administration. In addition, community events were held that encouraged project engagement through events like “Pastries with Pam,” “Light Lunch with Lucy”, “Java with Jon”, and every-semester “Staff & Faculty” and “Student” Feedback Sessions.

*Time*. Formal coursework in the URBAN project consisted of 16 credit hours per year (approximately 200 h/yr). Outside coursework, programming for skill development centered on workshops (15 h/yr), but also included meetings (3 h/yr) and a symposium or retreat (2 h/yr). The focal skills were outreach (15 h/yr), job readiness (13 h/yr), and communication for non-academic audiences (12 h/yr). Also important were communication to academic audiences (8 h/yr), team science and collaboration (7 h/yr), interdisciplinary research (4 h/yr), and critical thinking (4 h/yr).

*Institutionalization.* A great deal of the URBAN graduate project has been institutionalized at BU with continued Ph.D. student and faculty participation. The Introductory Workshop, City Governance, Science Communication, and Professional Development workshops, as well as the internship program, remain in place. URBAN’s Colloquium and Internship Companion courses also continue to be held.

*Challenges.* Challenges for the URBAN project include (1) engaging students, staff, and faculty across multiple departments that each have their own culture, norms, and requirements, (2) providing coursework that are effective for students coming from different backgrounds and capabilities, (3) integration across formal graduate requirements (e.g., dissertations) and interdisciplinary work for individual students, and (4) sustainability of the project after the end of the NSF NRT grant [28].

## Discussion

In this following discussion, we dive deeper into the 20 NRT projects and the three highlighted NRT projects. We also go into further details about the challenges posed to the three NRT projects and potential solutions for all NRT projects, NSF, and any institution that would like to adopt NRT-similar activities.

### Successes: Insights from the 20 NRT projects and the 3 NRT projects

Our examination of 20 NRT projects demonstrates various ways that professional development opportunities can be created for Trainees to learn technical and transferable skills that are frequently absent in traditional, discipline-based graduate programs. Although the activities of these projects overlap with activities that may be found in traditional programs (e.g., meetings, seminars), the innovation lies with the breadth of strategies, skill types, and topics that were used to train students in collaborative approaches whether it was in pursuit of team-based science or the co-development of knowledge with stakeholders.

It is important to note that we assessed these NRT projects prior to the recent federal and national emphasis on Artificial Intelligence (AI) and its many applications across multiple fields and problems. The recent 2025 cohort of newly funded NRT projects included a large swath of AI related goals with a focus on research training, which will bring a new dimension to graduate training that is not captured in this study. Regardless of project focus, the innovative aspects of the NRTs remain key to fostering interdisciplinary skills. At the same time, they are not free of challenges, one of which is the cost.

Across our three projects, we created new courses, symposium, internships, a graduate certificate, and a micro-credential. Perhaps more importantly, we created training and networking opportunities for faculty to aid in their interdisciplinary development and to provide space to build an interdisciplinary culture. We acknowledge that the development and integration of these types of activities, and its institutionalization, can be financially burdensome (e.g., time, labor). The costs of developing new coursework were incurred during the period in which federal funding was available. In contrast, each of our three NRTs sought additional funding to cover various elements, particularly the symposium.

Awardees and their institutions, our projects included, must make decisions about which components will continue beyond the lifetime of the project, and each project is responsible for selecting and securing support for those activities. We found that it was, indeed, possible to retain aspects of our programming. Implementation of new courses after the project’s sunset, for instance, were at a much lower cost to the faculty and the institution, and thus required no additional support. In contrast, each of our NRTs established public-private partnerships and on-campus commitments to sustain the annual symposia, demonstrating that sustainability of a costly activity is possible (Table 2). There are many other ways to institutionalize elements of a NRT, at low cost, but it takes creativity, commitment, and concerted effort to sustain implementation beyond the lifetime of an NRT.

### Challenges: Observations from the three NRT projects

The Climate Resilience, IMPACTS, and URBAN projects experienced several common sources of friction. The first challenge includes limits on faculty buy-in (e.g., engagement, contribution, management, leadership). Most faculty lack the knowledge and expertise necessary to conduct interdisciplinary work and to integrate “information, data, techniques, tools, perspectives, concepts or theories from two or more disciplines or bodies of specialized knowledge” [27,29]. While our three universities–Auburn, Boston, Michigan State–have an increasing array of opportunities to engage others, faculty often lack an appropriate network for making interdisciplinary connections and facilitating interdisciplinary education. This relationship building is compounded by the fact that faculty are time-poor. Most have a multitude of responsibilities and priorities, and when institutional team science training is offered, it competes with other job demands.

A second challenge is that the academic units remain siloed. One such example is the tenure system that remains firmly committed to individual excellence and discipline-based contributions, which creates challenges for early career researchers interested in interdisciplinary collaborations. This isolation is further maintained, in part, by universities’ use of funding mechanisms that create competition among colleges and departments for students and proposal funding, while also creating perverse incentives to offer similar coursework or research programs. These types of competitive boundaries can disincentivize inter-unit activities and services and impact the success of NRT projects more broadly.

In addition to unit funding, common organization and governance structures are also challenging. Antiquated facilities and administrative practices can stymie both the faculty’s willingness to participate in novel endeavors and their ability to obtain the necessary approval to develop cross-disciplinary ways of training graduate students. Such an example might include, a faculty member justifying to their administrator the time away from their home department to participate in a cross-unit project, but the administrator disapproves because the home department may not see a direct benefit from their efforts, even if it helps the institution overall. This same faculty member may need to be promoted and tenured in their home department, so if there is a lack of approval from higher administrators, they will be de-incentivized to participate.

Yet another challenge is rooted in policy and the larger community of science itself. For instance, while change has occurred, much of the science funding system continues to favor disciplinary-based research. Some exceptions (e.g., NSF Growing Convergence Research) exist, but most projects do not set explicit expectations for documenting knowledge and methods integration, nor do they provide documentation of best practices or capacity building opportunities at the institutional or administrative level. Consequently, projects focused on interdisciplinarity, transdisciplinary, and convergence science are likely to lack long-term organizational support.

### Solutions: Interdisciplinary graduate programs

Overcoming the challenges to interdisciplinarity requires a fundamental shift in how we support research that is aligned with national priorities and how we prepare and equip graduate students to be effective participants and future leaders in the process. Many activities used by the participating NRT projects do not require external input to implement or adapt. Interdisciplinary training and professional development can be introduced as part of an existing class in the form of a dedicated session, a formalized activity, or even a course redesign. Courses can also include team-based components in which students from different disciplines and backgrounds are intentionally matched. In cases where there is insufficient expertise to implement similar professional development activities, the diverse NRT projects are excellent points of contact to acquire materials, expertise, or even panelists and speakers.

The body of literature that assesses graduate programs, particularly the impact of interdisciplinary education, is quite limited as compared to undergraduate studies. Measuring the impact will require a nuanced and contextual approach. As interdisciplinarity programs continue to expand, it will become important to engage with educational and evaluation specialists. These experts bring the skills needed to assess the degree to which students and other stakeholders are advancing in their ability to conceptualize and develop solutions for complex socio-ecological problems [30]. Findings from our analysis suggests that it is, indeed, feasible to move beyond traditional disciplinary-based content, to shape long-lasting change in graduate education, and to prepare young scholars to join the professional workforce. Similar findings from recent work have shown that NRT projects can serve as curricular models in preparing graduate students to effectively communicate complicated and abstract scientific concepts to diverse audiences and engage with stakeholders [5]. Despite the challenges faced, we found the Trainees in our three projects to be excited about interdisciplinarity and the career outcome-based foci. They were receptive to new approaches and out-of-box thinking. We believe these students will serve as a conduit between disciplinary faculty and they will be capable of bringing new methods and new ways of thinking from other fields to their future research groups.

### Solutions: Funder and university engagement

*Project Sustainability***.** As NRT projects come to an end, at least some elements of each project need to be institutionalized. This is challenging for a number of reasons. For example, some program components may not be aligned with existing policies, structures, or available resources across departments making it difficult to formalize them. NSF ought to provide support in this process. For instance, NSF Program managers could conduct periodic in-person or virtual meetings not only with the project PIs, but also the institutional leadership (i.e., deans, provost, department chairs) at certain intervals. This interaction could help to create opportunities for shared decision-making and a structure for continuous communication within each institution that houses an NRT project [30]. This intentional communication would improve not only the alignment among departmental/college-level incentives and NRT Program elements, but also the promotion of solutions to any of the numerous challenges that NRT projects face. While we recognize the additional burden on NSF, which currently operates with an increasingly limited number of staff, we also see its potential to increase the overall efficiency and effectiveness of the NRT at large. As an alternative, the NRT solicitation and associated reporting systems could include specific requirements at the institutional level that incorporate administrator/NRT leadership collaboration.

*Program Scaling and Institutionalization.* With reduced congressional budgets to NSF and specifically educational programs, it would also be helpful to re-imagine how the Academy as a whole supports NRT activities. For example, integration of NRT-like activities into traditional research programs through funder required broader impact plans could be supported within existing and new funding calls. Additionally, continued NSF support for existing and new NRT projects through cross-project level supplements and annual Principal Investigator meetings to facilitate the formation of cross-institution collaborations and increasing interaction between these projects, where shared project goals and evaluation strategies, and lessons learned are communicated should occur. Such activities would serve to increase the knowledge base around effective NRT project implementation, associated efficacy, and increase the reach of NRT similar activities beyond NRT funded projects.

*Project Evaluation.* Although each NRT is required to retain their own external/internal evaluator and a rigorous evaluation plan, most NRT projects do not publish the findings of their external project evaluation. As such, the information collected about the efficacy and effectiveness of the individual projects beyond the required NSF reporting system, is lost. We recommend that NSF strongly encourage each project to disseminate the results of their educational interventions, alongside their interdisciplinary research findings.

The NRT Program overall is a catalyst to create change in graduate education across the U.S. To make impacts on an education system that has been held fairly constant over a time span of more than one-hundred years is not an outcome that will be achieved quickly. However, this paper illustrates the successes of these projects including a vast number of activities and educational innovations from a sub-sample of NRT projects aimed to enhance interdisciplinarity and workforce development in STEM. It also showcases the similarities and differences between three NRT projects, the challenges they faced and the potential solutions to address them. The Trainees and the graduate students that participate in NRT projects have been reported to greatly benefit from their NRT experiences. They are well-equipped and prepared for the workforce and are supported in gaining the skills and knowledge to solve interdisciplinary problems and communicate effectively, among several other documented benefits [5–7,28]. As a nation that has a great need to develop future STEM leaders, we should continue to support the NRT Program and its funded projects, continue to be creative in institutionalizing NRT project elements beyond their funding timeline, and propagate NRT-like elements throughout graduate education.

## Materials and methods

To investigate how contemporary NRT projects might serve as a model for innovative graduate education and interdisciplinary training, we requested professional development data from the currently funded projects. Drawing on the Tailored Design Method [31], an email request was sent to the respective project coordinators (N = 100) and repeated at 1 week and 3 weeks. Data were obtained from 20 NRT projects (i.e., 20% response rate) who provided their 2021 or 2022 project report Table of Professional Development (PD) activities that were developed as part of their NSF reporting requirements (see S1 Table). Complete annual reports could not be requested as they contain confidential and sensitive project data (e.g., participant names, demographics, and sensitive information about potential programmatic and budget changes). The Tables varied in length from single page descriptions to numerous pages outlining the details of their project activities. However, all were formatted in the same way and included the NSF suggested table elements (e.g., project’s training activities, professional development skills included, targeted stakeholders, delivery methods, time spent on the activity or skill). No additional information from the annual reports beyond these tables were provided for this study. The participating projects (see S2 Table) were from 12 states and included 16 public and 4 private institutions with doctoral programs. Of these projects, 16 were universities with very high research activity (R1) and 4 were universities with high research activity (R2). Most of the projects were located in the Midwest and Northeast. These projects mainly addressed the NSF Big Ideas of *Innovations at the Nexus of Food, Energy and Water Systems (INFEWS)* or the *Harnessing the Data Revolution for the 21st Century Science and Engineering (HDR)* and include topical and interdisciplinary areas such as environmental resilience and climate, robotics and autonomous systems and public policy, computational science and plant biology, urban communities and biogeosciences, astrophysics and electrical engineering, marine science and policy, human-robot interactions, among many others.

### Coding

We quantified the project data using a coding rubric (see S3 Table). The rubric was developed among the authorship team. It categorized each project’s activities (e.g., webinar, course, seminar), professional development skills (e.g., team science, communication, interdisciplinarity), targeted stakeholders (e.g., funded NRT Trainees, unfunded Trainees, graduate students), delivery methods (e.g., face to face, virtual), and time on activity or skill (e.g., number and duration of meetings per semester). Coding was conducted over multiple rounds. The first step required each author to test the rubric using the same project table, then the authors met as a team to discuss the results and make adjustments to the coding rubric (see S3 Table). Second, two coders assessed each project, and then the team met again to discuss and adjust the rubric. Third, each project was assigned two new coders for a third round of coding. The fourth step was to compare the codes to identify inconsistencies. To resolve inconsistencies another new coder was assigned to make the final code decision (i.e., tiebreaker). Thus, each project was coded multiple times by multiple coders with the goal of reaching 100% agreement.

### Analysis

Our analysis focused on three factors: activity descriptions (ActDes), professional skills (ProdSkill), and the stakeholders affected (StkSrvd, see S4 Table). For each of these three factors, the following eight frequencies were determined (see S4 Table): (1) count-ID-FACTOR: Counts of each code (e.g., 1: Meeting, columns) per institution ID (rows), (2) hr_year-FACTOR: Estimated total hours per year (column) for each code (row), (3) hr_year_inst-FACTOR: Estimated total hours per year for each code (column) per institution (row), (4) num_indiv-FACTOR: TNumPart, NumFun, NumNFun, and NumNTrain totals (columns) for each code (row), (5) TNumPart_inst-Factor: per institution version for TNumPart, (6) NumFun_inst-Factor: per institution version for NumFun, (7) NumNFun_inst-Factor: per institution version for NumNFun, (8) NumNTrain_inst-Factor: per institution version for NumNTrain. In terms of frequency, coding values were treated as follows: 2 - 1-2/week, assuming 15 weeks/semester, 3 - 1-2/month, assuming 12 months/year; 4 - 1-2/semester; 5 - 1-2/year, 88 and 99 – non-recurring. In addition to analyses of individual factors, frequencies of three possible factor pairs (ActDes-ProfSkill, StkSrvd-ProfSkill, ActDes-StkSrvd, S4 Table) were also determined. Analysis was conducted using a Python coded script (see S5 Table) on the final dataset (see S6 Table).

## Supporting information

S1 Table Table Headers from Annual Project Reports. (XLSX)

S2 Table NRT projects. (XLSX)

S3 Table Codebook to Analyze Report Table. (XLSX)

S4 Table Analysis. (DOCX)

S5 Table Code script. (DOCX)

S6 Table Anonymized Data Set from Study. (XLSX)
