# Supplementary material for: Lessons learned from interdisciplinary US national science foundation research traineeship-supported graduate programs
Source: PLoS One. 2026 Feb 20;21(2):e0343307. doi: 10.1371/journal.pone.0343307 (PMC12923050; doi:10.1371/journal.pone.0343307)
Supplement: S4 Table — (DOCX) [file pone.0343307.s004.docx]

The data used for the analysis is from the file Anonymized Data Set from Study.

### ***Individual factor analysis***

The three main factors focused on in the analysis are ActDes, ProfSkill, and StkSrvd. For each of these main factors the following analyses were conducted:

1. count-ID-FACTOR: Counts of each code (e.g., 1: Meeting, columns) per institution ID (rows)
2. hr_year-FACTOR: Estimated total hours per year (column) for each code (row)
3. hr_year_inst-FACTOR: Estimated total hours per year for each code (column) per institution (row)
4. num_indiv-FACTOR: TNumPart, NumFun, NumNFun, and NumNTrain totals (columns) for each code (row)
5. TNumPart_inst-Factor: per institution version for TNumPart
6. NumFun_inst-Factor: per institution version for NumFun
7. NumNFun_inst-Factor: per institution version for NumNFun
8. NumNTrain_inst-Factor: per institution version for NumNTrain

Some assumptions were made when estimating time. These include:

# estimate per year, factor to multiply with

freq_dict = {1: 1, # non-recurring

2: 1.5*15, # 1-2/week, assuming 15 weeks/semester, one per year

3: 1.5*12, # 1-2/month, assuming 12 months/year

4: 1.5*2, # 1-2/semester

5: 1.2*1, # 1-2/year

88: 1, # treat as non-recurring

99: 1 # treat as non-recurring

}

### ***Joint factor analysis***

Pairwise comparisons were done between two of the three focal factors above.
