# Supplementary material for: Lessons learned from interdisciplinary US national science foundation research traineeship-supported graduate programs
Source: PLoS One. 2026 Feb 20;21(2):e0343307. doi: 10.1371/journal.pone.0343307 (PMC12923050; doi:10.1371/journal.pone.0343307)
Supplement: S5 Table — (DOCX) [file pone.0343307.s005.docx]

{

"cells": [

{

"cell_type": "markdown", "metadata": {},

"source": [

"# NRT professioal development analysis \n", "\n",

"\n",

"Log:\n",

"- 10/30/23\n",

" - Resume analysis\n", "- 10/22/23\n",

" - Coding error corrected by McNeal \n", "10/14/23\n",

"- Preprocess data"

]

},

{

"cell_type": "markdown", "metadata": {},

"source": [

"## Setup "

]

},

{

"cell_type": "code", "execution_count": 1, "metadata": {},

"outputs": [], "source": [

"import pandas as pd\n", "import numpy as np\n", "from pathlib import Path"

]

},

{

"cell_type": "code", "execution_count": 2, "metadata": {},

"outputs": [], "source": [

"work_dir = Path.home() / 'proj_onedrive/nrt'\n", "excel_file = work_dir / 'tie_break_coding_round4.xlsx'\n", "data_sheet = \"tiebreak combine 10-30\"\n",

"code_sheet = \"coding_for_analysis\""

]

},

{

"cell_type": "markdown", "metadata": {},

"source": [

"## Preprocess file "

]

},

{

"cell_type": "markdown", "metadata": {},

"source": [

"### Read in the data"

]

},

{

"cell_type": "code", "execution_count": 3, "metadata": {}, "outputs": [

{

"data": {

"text/html": [ "<div>\n",

"<style scoped>\n",

" .dataframe tbody tr th:only-of-type {\n", " vertical-align: middle;\n",

" }\n",

"\n",

" .dataframe tbody tr th {\n", " vertical-align: top;\n",

" }\n",

"\n",

" .dataframe thead th {\n", " text-align: right;\n",

" }\n", "</style>\n",

"<table border=\"1\" class=\"dataframe\">\n", " <thead>\n",

" <tr style=\"text-align: right;\">\n", " <th></th>\n",

" <th>Coder</th>\n", " <th>ID</th>\n",

" <th>Row</th>\n",

" <th>ActDes</th>\n", " <th>ProfSkill</th>\n", " <th>MthDel</th>\n", " <th>Length</th>\n",

" <th>Freq</th>\n",

" <th>ReqOpt</th>\n", " <th>StkSrvd</th>\n",

" <th>TNumPart</th>\n", " <th>NumFun</th>\n",

" <th>NumNFun</th>\n", " <th>NumNTrain</th>\n", " </tr>\n",

" </thead>\n", " <tbody>\n", " <tr>\n",

" <th>0</th>\n",

" <td>McNeal</td>\n", " <td>5</td>\n",

" <td>1</td>\n", " <td>2</td>\n",

" <td>7,3,1</td>\n", " <td>2</td>\n",

" <td>75</td>\n", " <td>1</td>\n", " <td>1</td>\n",

" <td>1,4</td>\n", " <td>14</td>\n", " <td>7</td>\n",

" <td>6</td>\n", " <td>0</td>\n", " </tr>\n",

" <tr>\n",

" <th>1</th>\n",

" <td>McNeal</td>\n", " <td>5</td>\n",

" <td>2</td>\n", " <td>2</td>\n",

" <td>11,8,12</td>\n", " <td>2</td>\n",

" <td>60</td>\n", " <td>1</td>\n", " <td>1</td>\n",

" <td>1,2,4</td>\n", " <td>14</td>\n",

" <td>6</td>\n", " <td>6</td>\n", " <td>1</td>\n", " </tr>\n",

" </tbody>\n", "</table>\n", "</div>"

],

"text/plain": [

" Coder ID Row ActDes ProfSkill MthDel Length Freq ReqOpt StkSrvd \\\n", "0 McNeal 5 1 2 7,3,1 2 75 1 1 1,4 \n",

"1 McNeal 5 2 2 11,8,12 2 60 1 1 1,2,4 \n",

"\n",

" TNumPart NumFun NumNFun NumNTrain \n", "0 14 7 6 0 \n",

"1 14 6 6 1 "

]

},

"execution_count": 3, "metadata": {},

"output_type": "execute_result"

}

],

"source": [

"data = pd.read_excel(excel_file, sheet_name=data_sheet)\n", "data.head(2)"

]

},

{

"cell_type": "code", "execution_count": 4, "metadata": {}, "outputs": [

{

"data": {

"text/html": [ "<div>\n",

"<style scoped>\n",

" .dataframe tbody tr th:only-of-type {\n", " vertical-align: middle;\n",

" }\n",

"\n",

" .dataframe tbody tr th {\n", " vertical-align: top;\n",

" }\n",

"\n",

" .dataframe thead th {\n", " text-align: right;\n",

" }\n", "</style>\n",

"<table border=\"1\" class=\"dataframe\">\n", " <thead>\n",

" <tr style=\"text-align: right;\">\n", " <th></th>\n",

" <th>Category</th>\n", " <th>Code</th>\n",

" <th>Desc</th>\n",

" <th>Additional details</th>\n", " </tr>\n",

" </thead>\n", " <tbody>\n", " <tr>\n",

" <th>0</th>\n",

" <td>ActDes</td>\n", " <td>1</td>\n",

" <td>Meeting</td>\n",

" <td>(not as formal, e.g., brainstorming, updating ...</td>\n", " </tr>\n",

" <tr>\n",

" <th>1</th>\n",

" <td>ActDes</td>\n", " <td>2</td>\n",

" <td>Workshop/panel/boot_camp</td>\n", " <td>NaN</td>\n",

" </tr>\n",

" </tbody>\n", "</table>\n", "</div>"

],

"text/plain": [

" Category Code Desc \\\n",

"0 ActDes 1 Meeting \n",

"1 ActDes 2 Workshop/panel/boot_camp \n", "\n",

" Additional details \n",

"0 (not as formal, e.g., brainstorming, updating ... \n", "1 NaN "

]

},

"execution_count": 4, "metadata": {},

"output_type": "execute_result"

}

],

"source": [

"code = pd.read_excel(excel_file, sheet_name=code_sheet)\n", "code.head(2)"

]

},

{

"cell_type": "markdown", "metadata": {},

"source": [

"### Check for data format"

]

},

{

"cell_type": "markdown", "metadata": {},

"source": [

"#### Create category dictoinary"

]

},

{

"cell_type": "code", "execution_count": 5, "metadata": {}, "outputs": [

{

"name": "stdout", "output_type": "stream", "text": [

"{'ActDes': {1: 'Meeting', 2: 'Workshop/panel/boot_camp', 3: 'Course', 4: 'NRT_seminar', 5:

'NRT_symposium_or_Conference', 6: 'Retreat/social_activities', 7: 'Internship', 8: 'Outreach_activity', 9: 'Other', 88:

'Unknown', 99: 'no_response_or_description'}, 'ProfSkill': {1: 'Training_in_interdisciplinary_skills', 2:

'Training_in_disciplinary_skills', 3: 'Critical_thinking', 4: 'Critical_thinking-ethics', 5:

'Team_science_&_collaboration_skill', 6: 'Teaching_&_Mentoring_', 7: 'Entrepreneurship_and_IP', 8:

'Communication_-_academic', 9: 'Communication_-_non-academic', 10: 'DEI/Broadening_participation', 11:

'Job_readiness', 12: 'Others', 13: 'Outreach', 88: 'Unknown'}, 'MthDel': {1: 'F2F', 2: 'Virtual', 3: 'Hybrid', 88: 'Unknown', 99: 'no_response_or_description'}, 'Length': {'<num>': nan, 888: 'Unknown', 999: 'no_response_or_description'}, 'Freq':

{1: 'Non-recurring', 2: '1-2/week', 3: '1-2/month', 4: '1-2/semester', 5: '1-2/year', 88: 'Unknown', 99:

'no_response_or_description'}, 'ReqOpt': {1: 'Required', 2: 'Optional', 88: 'Unknown', 99: 'no_response_or_description'},

'StkSrvd': {1: 'Trainees', 2: 'Other_graduate_students', 3: 'Faculty', 4: 'Program_Coordinator/staff', 5:

'Advisory_Committee/evaluator', 6: 'Organization_offerring_internship', 7: 'Partner_organization', 8:

'Public_stakeholders', 9: 'Others', 88: 'Unknown', 99: 'no_response_or_description'}, 'TNumPart': {'<num>': nan, 777:

'Others', 888: 'Unknown', 999: 'no_response_or_description'}, 'NumFun': {'<num>': nan, 888: 'Unknown', 999: 'no_response_or_description'}, 'NumNFun': {'<num>': nan, 888: 'Unknown', 999: 'no_response_or_description'}, 'NumNTrain': {'<num>': nan, 777: 'Others', 888: 'Unknown', 999: 'no_response_or_description'}}\n"

]

}

],

"source": [

"cat_dict = {} # {category: {code: short_desc}}\n", "cat_list = code['Category'].values.tolist()\n", "code_list = code['Code'].values.tolist()\n", "desc_list = code['Desc'].values.tolist()\n",

"for idx, cat in enumerate(cat_list):\n", " code = code_list[idx]\n",

" desc = desc_list[idx]\n", " if cat not in cat_dict:\n",

" cat_dict[cat] = {code:desc}\n", " else:\n",

" cat_dict[cat][code] = desc\n", "\n",

"print(cat_dict)"

]

},

{

"cell_type": "markdown", "metadata": {},

"source": [

"#### Check each category"

]

},

{

"cell_type": "code", "execution_count": 6, "metadata": {}, "outputs": [

{

"data": { "text/plain": [

"Index(['ActDes', 'ProfSkill', 'MthDel', 'Length', 'Freq', 'ReqOpt', 'StkSrvd',\n", " 'TNumPart', 'NumFun', 'NumNFun', 'NumNTrain'],\n",

" dtype='object')"

]

},

"execution_count": 6, "metadata": {},

"output_type": "execute_result"

}

],

"source": [

"target_cat = data.columns[3:]\n", "target_cat"

]

},

{

"cell_type": "code", "execution_count": 7, "metadata": {}, "outputs": [

{

"data": { "text/plain": [

"{1: 'Required', 2: 'Optional', 88: 'Unknown', 99: 'no_response_or_description'}"

]

},

"execution_count": 7, "metadata": {},

"output_type": "execute_result"

}

],

"source": [ "cat_dict['ReqOpt']"

]

},

{

"cell_type": "code", "execution_count": 8, "metadata": {}, "outputs": [

{

"data": { "text/plain": [

"Index(['Coder', 'ID', 'Row', 'ActDes', 'ProfSkill', 'MthDel', 'Length', 'Freq',\n",

" 'ReqOpt', 'StkSrvd', 'TNumPart', 'NumFun', 'NumNFun', 'NumNTrain'],\n", " dtype='object')"

]

},

"execution_count": 8, "metadata": {},

"output_type": "execute_result"

}

],

"source": [ "data.columns"

]

},

{

"cell_type": "code", "execution_count": 9, "metadata": {}, "outputs": [

{

"name": "stdout", "output_type": "stream", "text": [

"ActDes\n", "ProfSkill\n", "MthDel\n", "Length\n",

"Freq\n", "ReqOpt\n", "StkSrvd\n", "TNumPart\n", "NumFun\n", "NumNFun\n", "NumNTrain\n"

]

}

],

"source": [

"# Go through each category\n", "for cat in target_cat:\n",

" print(cat)\n",

" vals = data[cat].values.tolist() # values for this category\n", "\n",

" # go through each value\n",

" for idx, val in enumerate(vals):\n", " COD = data['Coder'][idx]\n",

" ID = data['ID'][idx]\n",

" ROW = data[\"Row\"][idx]\n", " \n",

" # if multiple values, split\n", " if type(val) == str:\n",

" val_list = val.split(\",\")\n", " try:\n",

" val_list = [int(v) for v in val_list]\n", " except ValueError:\n",

" print(f\" ERR 3: coder={COD}, id={ID}, row={ROW}, val_list={val_list}\")\n", " val_list = []\n",

" else:\n",

" val_list = [val]\n", "\n",

" # Check values\n", " for v in val_list:\n", " err = 0\n",

" # Need int or float\n",

" if \"<num>\" in cat_dict[cat]:\n",

" if type(v) != int and type(v) != float:\n", " err = 1\n",

" # already is a value\n",

" elif v not in cat_dict[cat]:\n", " err = 2\n",

" if err != 0:\n",

" print(f\" ERR {err}: coder={COD}, id={ID}, row={ROW}, val={v}\")\n"

]

},

{

"cell_type": "markdown", "metadata": {},

"source": [

"## Common functions "

]

},

{

"cell_type": "markdown", "metadata": {},

"source": [

"### Joint two columns and count unique combinations"

]

},

{

"cell_type": "code", "execution_count": 10, "metadata": {},

"outputs": [], "source": [

"def get_val_list(val):\n", " if type(val) == str:\n",

" v_list = val.split(\",\")\n",

" v_list = [int(v) for v in v_list]\n", " else:\n",

" v_list = [val]\n", "\n",

" return v_list"

]

},

{

"cell_type": "code", "execution_count": 11, "metadata": {},

"outputs": [], "source": [

"def joint_two_columns(data, cat_dict, col1, col2, col1_is_iid=0):\n",

" '''Count the number of occurences for a col1-col2 value combination\n", " Args:\n",

" data (pd.DataFrame): dataframe with prof dev coding info\n", " cat_dict(dict): {category: {code: short_desc}}\n",

" col1 (str): column name 1\n", " col2 (str): column name 2\n",

" col1_is_iid (int): 1 if col1 is institutional ID, 0 otherwise\n", " Return:\n",

" dict_c1_c2 (dict): {col1 value: {col2 value: count}}\n",

" df (pd.DataFrame): dataframe with col1 idx as rows and col2 idx as columns, \n", " counts as values, added last column and row for marginal total (for the\n",

" cells, if val > 0, set to 1, if val == 0, set to 0)\n", " Output\n",

" tsv file: with col1 idx as rows and col2 idx as columns, counts as values\n", " '''\n",

" # Number of institutions offerring\n",

" ser1 = data[col1] # e.g, institutional ID\n", " ser2 = data[col2] # e.g, activities\n",

" if col1_is_iid:\n",

" idx_ser1 = np.unique(data[\"ID\"].values)\n", " else:\n",

" idx_ser1 = list(cat_dict[col1].keys())\n", "\n",

" idx_ser2 = list(cat_dict[col2].keys())\n",

" idx_ser1.sort()\n", " idx_ser2.sort()\n", "\n",

" dict_c1_c2 = {} # e.g., {institutional ID: {activity idx: count}}\n", " for idx, ser2_val in enumerate(ser2):\n",

"\n",

" # Check if there are multiple codes for series 1\n", " s1_list = get_val_list(ser1[idx])\n",

" \n",

" # Check if there are multiple codes for series 2\n", " s2_list = get_val_list(ser2_val)\n",

"\n",

" # Get count\n",

" for v2 in s2_list:\n", " for v1 in s1_list:\n",

" if v1 not in dict_c1_c2:\n",

" dict_c1_c2[v1] = {v2:1}\n",

" elif v2 not in dict_c1_c2[v1]:\n", " dict_c1_c2[v1][v2] = 1\n",

" else:\n",

" dict_c1_c2[v1][v2] += 1\n", "\n",

" # Fill in 0s\n",

" # For series 1, in case there is no value for a series1 index\n", " for idx1 in idx_ser1:\n",

" if idx1 not in dict_c1_c2:\n", " dict_c1_c2[idx1] = {}\n", "\n",

" # For series 2 \n",

" for idx2 in dict_c1_c2:\n",

" ser2_dict = dict_c1_c2[idx2]\n", " for idx in idx_ser2:\n",

" if idx not in ser2_dict:\n", " ser2_dict[idx] = 0\n", "\n",

" # Convert dict to dataframe\n",

" df = pd.DataFrame(dict_c1_c2).T # transpose so col1 is row index\n", " df.sort_index(axis=0, inplace=True) # sort by col1\n",

" df.sort_index(axis=1, inplace=True) # sort by col2\n", "\n",

" # Add index column name\n", " df.index.name = col1\n",

"\n",

" # Rename columns\n",

" df.columns = [f\"{idx}_{cat_dict[col2][idx]}\" for idx in df.columns]\n", "\n",

" # Rename rows if not institutional ID\n", " if not col1_is_iid:\n",

" df.index = [f\"{idx}_{cat_dict[col1][idx]}\" for idx in df.index]\n", "\n",

" return dict_c1_c2, df"

]

},

{

"cell_type": "markdown", "metadata": {},

"source": [

"### Detetrmine duration\n", "\n",

"- 10/30/23\n",

" - E.g. hr_per_year-ActDes, the values do not seem right as the value per institution is a lot higher than this."

]

},

{

"cell_type": "code", "execution_count": 12, "metadata": {},

"outputs": [], "source": [

"def time_spent(data, cat_dict, col1):\n",

" '''Time spend on different activities, skills, or stackholders\n", " Args:\n",

" data (pd.DataFrame): dataframe with prof dev coding info\n", " cat_dict(dict): {category: {code: short_desc}}\n",

" col1 (str): column name to determine time spent\n", " Return:\n",

" dict_total_time (dict): {col1 value: [col_name, num_freq_unk, num_len_unk, \n", " total_time_in_hour]}\n",

" df_total_time (pd.DataFrame): dataframe converted from dict_total_time\n", " '''\n",

" # estimate per year, factor to multply with\n", " freq_dict = {1: 1, # non-recurring\n",

" 2: 1.5*15, # 1-2/week, assuming 15 weeks/semester, one per year\n", " 3: 1.5*12, # 1-2/month, assuming 12 months/year\n",

" 4: 1.5*2, # 1-2/semester\n",

" 5: 1.2*1, # 1-2/year\n",

" 88: 1, # treat as non-recurring\n",

" 99: 1 # treat as non-recurring\n", " }\n",

"\n",

" # {col1: [col_name, num_freq_unk, num_len_unk, total_time]}\n", " dict_total_time = {}\n",

" for idx in data.index:\n",

" val_col = data.loc[idx, col1]\n", "\n",

" if isinstance(val_col, int):\n", " val_col = str(val_col)\n", "\n",

" for val in val_col.split(\",\"):\n", " val = int(val)\n",

" name_col = cat_dict[col1][val]\n", "\n",

" if val not in dict_total_time:\n",

" dict_total_time[val] = [name_col, 0, 0, 0]\n", "\n",

" val_len = data.loc[idx, \"Length\"]\n", " val_freq = data.loc[idx, \"Freq\"]\n", "\n",

" flag_unk = 0\n",

" if val_freq == 88 or val_freq == 99:\n", " dict_total_time[val][1] += 1\n",

" if val_len == 888 or val_freq == 999:\n", " dict_total_time[val][2] += 1\n",

" flag_unk = 1\n", "\n",

" if not flag_unk:\n",

" t = val_len * freq_dict[val_freq]\n", " dict_total_time[val][3] += t/60\n", " \n",

" # Convert dict to dataframe\n",

" df = pd.DataFrame(dict_total_time).T # transpose so col1 is row index\n", "\n",

" df.columns = [col1, \"num_freq_unk\", \"num_len_unk\", \"total_time (hr/year)\"]\n", " df.sort_index(axis=0, inplace=True) # sort by rows\n",

"\n",

" return dict_total_time, df\n", "\n"

]

},

{

"cell_type": "code", "execution_count": 13, "metadata": {},

"outputs": [], "source": [

"def time_spent_per_inst(data, cat_dict, col1):\n",

" '''Time spend on different activities, skills, or stackholders per institution\n", " Args:\n",

" data (pd.DataFrame): dataframe with prof dev coding info\n", " cat_dict(dict): {category: {code: short_desc}}\n",

" col1 (str): column name to determine time spent\n", " Return:\n",

" dict_iid_time (dict): {iid: {cat: time_in_hour}}\n",

" df_iid_time (pd.DataFrame): dataframe converted from dict_iid_time\n", " '''\n",

"\n",

" # catergories\n",

" cats = list(cat_dict[col1].keys())[:-1]\n", "\n",

" dict_iid_time = {} # {iid: {cat: time}}\n", " for iid in range(1,21):\n",

" dict_iid_time[iid] = {}\n", "\n",

" # Get data subset for each institution\n", " data_iid = data[data[\"ID\"] == iid]\n", "\n",

" #if iid == 19: print(data_iid)\n", "\n",

" # Get {col1 value: [col_name, num_freq_unk, num_len_unk, total_time_in_hr]}\n", " dict_time_spent_iid, _ = time_spent(data_iid, cat_dict, col1)\n",

"\n",

" #if iid == 19: print(dict_time_spent_iid)\n",

"\n",

" # Go through each category and fill values\n", " for cat in cats:\n",

" if cat in dict_time_spent_iid:\n",

" dict_iid_time[iid][cat] = dict_time_spent_iid[cat][3]\n", " else:\n",

" dict_iid_time[iid][cat] = 0\n", "\n",

" # Convert to df, with 1 decimal place\n",

" df_iid_time = pd.DataFrame(dict_iid_time).T.round(1)\n", "\n",

" return dict_iid_time, df_iid_time\n", " "

]

},

{

"cell_type": "code", "execution_count": 14, "metadata": {},

"outputs": [], "source": [

"def get_num_indiv(data, cat_dict, col1):\n",

" '''Coiunt the number of individuals involved'''\n", "\n",

" # {col1: [col_name, TNumPart,\tNumFun,\tNumNFun,\tNumNTrain]}\n", " dict_total_indiv = {}\n",

" for idx in data.index:\n",

" val_col = data.loc[idx, col1]\n", "\n",

" if isinstance(val_col, int):\n", " val_col = str(val_col)\n", "\n",

" for val in val_col.split(\",\"):\n", " val = int(val)\n",

" name_col = cat_dict[col1][val]\n", "\n",

" if val not in dict_total_indiv:\n",

" dict_total_indiv[val] = [name_col, 0, 0, 0, 0]\n", "\n",

" num_indiv = [data.loc[idx, \"TNumPart\"],\n", " data.loc[idx, \"NumFun\"],\n",

" data.loc[idx, \"NumNFun\"],\n",

" data.loc[idx, \"NumNTrain\"]]\n", " \n",

" # Set value to 0 if 777, 888, or 999\n",

" num_indiv = [n if n not in [777, 888, 999] else 0 for n in num_indiv]\n", "\n",

" # Add to list\n",

" dict_total_indiv[val][1] += num_indiv[0]\n", " dict_total_indiv[val][2] += num_indiv[1]\n", " dict_total_indiv[val][3] += num_indiv[2]\n", " dict_total_indiv[val][4] += num_indiv[3]\n", "\n",

" # Convert dict to dataframe\n",

" df = pd.DataFrame(dict_total_indiv).T # transpose so col1 is row index\n", "\n",

" df.columns = [col1, \"TNumPart\", \"NumFun\", \"NumNFun\", \"NumNTrain\"]\n", " df.sort_index(axis=0, inplace=True) # sort by rows\n",

"\n",

" return dict_total_indiv, df\n"

]

},

{

"cell_type": "code", "execution_count": 15, "metadata": {},

"outputs": [], "source": [

"def get_num_indiv_per_inst(data, cat_dict, col1):\n",

" '''Coiunt the number of individuals involved per institution\n", " Args:\n",

" data (pd.DataFrame): dataframe with prof dev coding info\n", " cat_dict(dict): {category: {code: short_desc}}\n",

" col1 (str): column name to determine time spent\n", " Return:\n",

" dict_iid_indiv_ntp (dict): total num participants/institution/col1 category\n", " dict_iid_indiv_nf (dict): num funded trainees/institution/col1 category\n",

" dict_iid_indiv_nnf (dict): num non-funded trainees/institution/col1 category\n", " dict_iid_indiv_nnt (dict): num non-trainees/institution/col1 category\n",

" '''\n",

"\n",

" # catergories\n",

" cats = list(cat_dict[col1].keys())[:-1]\n", "\n",

" # {iid: {cat: num_indiv}\n",

" dict_iid_indiv_ntp = {} # total number of participants\n", " dict_iid_indiv_nf = {} # number of funded trainees\n",

" dict_iid_indiv_nnf = {} # number of non-funded trainees\n", " dict_iid_indiv_nnt = {} # number of non-trainees\n",

"\n",

" # Go through institutions\n", " for iid in range(1,21):\n",

" dict_iid_indiv_ntp[iid] = {}\n", " dict_iid_indiv_nf[iid] = {}\n", " dict_iid_indiv_nnf[iid] = {}\n", " dict_iid_indiv_nnt[iid] = {}\n", "\n",

" # Get data subset for each institution\n", " data_iid = data[data[\"ID\"] == iid]\n", "\n",

" # Get {col1: [col_name, TNumPart,\tNumFun,\tNumNFun,\tNumNTrain]} for iid\n", " dict_total_indiv_iid, _ = get_num_indiv(data_iid, cat_dict, col1)\n",

"\n",

" # Go through each category and fill values\n", " for cat in cats:\n",

" if cat in dict_total_indiv_iid:\n",

" dict_iid_indiv_ntp[iid][cat] = dict_total_indiv_iid[cat][1]\n", " dict_iid_indiv_nf[iid][cat] = dict_total_indiv_iid[cat][2]\n",

" dict_iid_indiv_nnf[iid][cat] = dict_total_indiv_iid[cat][3]\n", " dict_iid_indiv_nnt[iid][cat] = dict_total_indiv_iid[cat][4]\n", " else:\n",

" dict_iid_indiv_ntp[iid][cat] = 0\n", " dict_iid_indiv_nf[iid][cat] = 0\n", " dict_iid_indiv_nnf[iid][cat] = 0\n", " dict_iid_indiv_nnt[iid][cat] = 0\n", "\n",

" # Convert to df\n",

" df_iid_indiv_ntp = pd.DataFrame(dict_iid_indiv_ntp).T\n", " df_iid_indiv_nf = pd.DataFrame(dict_iid_indiv_nf).T\n",

" df_iid_indiv_nnf = pd.DataFrame(dict_iid_indiv_nnf).T\n", " df_iid_indiv_nnt = pd.DataFrame(dict_iid_indiv_nnt).T\n", "\n",

" return df_iid_indiv_ntp, df_iid_indiv_nf, df_iid_indiv_nnf, df_iid_indiv_nnt"

]

},

{

"cell_type": "markdown", "metadata": {},

"source": [

"## Analysis: activity \n", "\n",

"- Number of institutions offerring\n", "- Fraction of total activities\n",

"- Total min per act per inst\n",

"- Act occurrence: # of institutions\n",

"- Numbers of total individuals impacted\n", "- Vs. prof skills\n",

"- Vs. stackholders"

]

},

{

"cell_type": "markdown", "metadata": {},

"source": [

"### Get ID-activity counts"

]

},

{

"cell_type": "code", "execution_count": 16, "metadata": {},

"outputs": [], "source": [

"# Count unique ID-ActDes combinations\n",

"dict_id_act, df_id_act = joint_two_columns(data, cat_dict, \"ID\", \"ActDes\", 1)"

]

},

{

"cell_type": "code", "execution_count": 17, "metadata": {}, "outputs": [

{

"data": {

"text/html": [ "<div>\n",

"<style scoped>\n",

" .dataframe tbody tr th:only-of-type {\n", " vertical-align: middle;\n",

" }\n",

"\n",

" .dataframe tbody tr th {\n", " vertical-align: top;\n",

" }\n",

"\n",

" .dataframe thead th {\n", " text-align: right;\n",

" }\n", "</style>\n",

"<table border=\"1\" class=\"dataframe\">\n", " <thead>\n",

" <tr style=\"text-align: right;\">\n", " <th></th>\n",

" <th>1_Meeting</th>\n",

" <th>2_Workshop/panel/boot_camp</th>\n", " <th>3_Course</th>\n",

" <th>4_NRT_seminar</th>\n",

" <th>5_NRT_symposium_or_Conference</th>\n", " <th>6_Retreat/social_activities</th>\n",

" <th>7_Internship</th>\n",

" <th>8_Outreach_activity</th>\n", " <th>9_Other</th>\n",

" <th>88_Unknown</th>\n",

" <th>99_no_response_or_description</th>\n", " </tr>\n",

" <tr>\n",

" <th>ID</th>\n", " <th></th>\n",

" <th></th>\n",

" <th></th>\n",

" <th></th>\n",

" <th></th>\n",

" <th></th>\n",

" <th></th>\n",

" <th></th>\n",

" <th></th>\n",

" <th></th>\n",

" <th></th>\n",

" </tr>\n",

" </thead>\n", " <tbody>\n", " <tr>\n",

" <th>1</th>\n", " <td>2</td>\n", " <td>2</td>\n", " <td>2</td>\n",

" <td>0</td>\n", " <td>0</td>\n", " <td>2</td>\n", " <td>0</td>\n", " <td>0</td>\n", " <td>0</td>\n", " <td>0</td>\n", " <td>0</td>\n", " </tr>\n",

" <tr>\n",

" <th>2</th>\n", " <td>7</td>\n", " <td>11</td>\n", " <td>6</td>\n", " <td>3</td>\n", " <td>1</td>\n", " <td>1</td>\n", " <td>0</td>\n", " <td>9</td>\n", " <td>7</td>\n", " <td>0</td>\n", " <td>0</td>\n", " </tr>\n",

" </tbody>\n", "</table>\n", "</div>"

],

"text/plain": [

" 1_Meeting 2_Workshop/panel/boot_camp 3_Course 4_NRT_seminar \\\n", "ID \n",

"1 2 2 2 0 \n",

"2 7 11 6 3 \n",

"\n",

" 5_NRT_symposium_or_Conference 6_Retreat/social_activities 7_Internship \\\n", "ID \n",

"1 0 2 0 \n",

"2 1 1 0 \n",

"\n",

" 8_Outreach_activity 9_Other 88_Unknown 99_no_response_or_description \n",

| "ID |  |  |  | \n", |
| --- | --- | --- | --- | --- |
| "1 | 0 | 0 | 0 | 0 \n", |
| "2 | 9 | 7 | 0 | 0 " |
| ] |  |  |  |  |

},

"execution_count": 17, "metadata": {},

"output_type": "execute_result"

}

],

"source": [ "df_id_act.head(2)"

]

},

{

"cell_type": "code", "execution_count": 18, "metadata": {}, "outputs": [

{

"data": {

"text/html": [ "<div>\n",

"<style scoped>\n",

" .dataframe tbody tr th:only-of-type {\n", " vertical-align: middle;\n",

" }\n",

"\n",

" .dataframe tbody tr th {\n", " vertical-align: top;\n",

" }\n",

"\n",

" .dataframe thead th {\n", " text-align: right;\n",

" }\n", "</style>\n",

"<table border=\"1\" class=\"dataframe\">\n", " <thead>\n",

" <tr style=\"text-align: right;\">\n", " <th></th>\n",

" <th>1_Meeting</th>\n",

" <th>2_Workshop/panel/boot_camp</th>\n", " <th>3_Course</th>\n",

" <th>4_NRT_seminar</th>\n",

" <th>5_NRT_symposium_or_Conference</th>\n", " <th>6_Retreat/social_activities</th>\n",

" <th>7_Internship</th>\n",

" <th>8_Outreach_activity</th>\n", " <th>9_Other</th>\n",

" <th>88_Unknown</th>\n",

" <th>99_no_response_or_description</th>\n", " </tr>\n",

" <tr>\n",

" <th>ID</th>\n", " <th></th>\n",

" <th></th>\n",

" <th></th>\n",

" <th></th>\n",

" <th></th>\n",

" <th></th>\n",

" <th></th>\n",

" <th></th>\n",

" <th></th>\n",

" <th></th>\n",

" <th></th>\n",

" </tr>\n",

" </thead>\n", " <tbody>\n", " <tr>\n",

" <th>19</th>\n", " <td>0</td>\n", " <td>2</td>\n", " <td>1</td>\n", " <td>0</td>\n", " <td>1</td>\n", " <td>0</td>\n", " <td>0</td>\n", " <td>1</td>\n", " <td>0</td>\n", " <td>0</td>\n", " <td>0</td>\n", " </tr>\n",

" <tr>\n",

" <th>20</th>\n", " <td>2</td>\n", " <td>3</td>\n", " <td>0</td>\n", " <td>5</td>\n", " <td>0</td>\n", " <td>1</td>\n", " <td>1</td>\n", " <td>1</td>\n", " <td>7</td>\n", " <td>0</td>\n", " <td>0</td>\n", " </tr>\n",

" </tbody>\n", "</table>\n", "</div>"

],

"text/plain": [

" 1_Meeting 2_Workshop/panel/boot_camp 3_Course 4_NRT_seminar \\\n",

| "ID |  |  |  | \n", |
| --- | --- | --- | --- | --- |
| "19 | 0 | 2 | 1 | 0 \n", |
| "20 | 2 | 3 | 0 | 5 \n", |
| "\n", |  |  |  |  |

" 5_NRT_symposium_or_Conference 6_Retreat/social_activities 7_Internship \\\n", "ID \n",

"19 1 0 0 \n",

"20 0 1 1 \n",

"\n",

" 8_Outreach_activity 9_Other 88_Unknown 99_no_response_or_description \n",

| "ID |  |  |  | \n", |
| --- | --- | --- | --- | --- |
| "19 | 1 | 0 | 0 | 0 \n", |
| "20 | 1 | 7 | 0 | 0 " |
| ] |  |  |  |  |

},

"execution_count": 18, "metadata": {},

"output_type": "execute_result"

}

],

"source": [

"df_id_act.tail(2)"

]

},

{

"cell_type": "markdown", "metadata": {},

"source": [

"### Activity time dedicated"

]

},

{

"cell_type": "code", "execution_count": 19, "metadata": {},

"outputs": [], "source": [

"dict_total_act, df_total_act = time_spent(data, cat_dict, \"ActDes\")"

]

},

{

"cell_type": "code", "execution_count": 20, "metadata": {}, "outputs": [

{

"data": { "text/plain": [

"{2: ['Workshop/panel/boot_camp', 9, 2, 276.2],\n",

" 6: ['Retreat/social_activities', 1, 1, 25.083333333333336],\n",

" 4: ['NRT_seminar', 4, 1, 199.25],\n",

" 3: ['Course', 0, 3, 1155.75],\n",

" 1: ['Meeting', 6, 3, 351.1],\n",

" 7: ['Internship', 1, 1, 0],\n",

" 9: ['Other', 9, 5, 179.25833333333333],\n",

" 8: ['Outreach_activity', 1, 1, 196.90833333333333],\n",

" 88: ['Unknown', 2, 0, 62.0],\n",

" 5: ['NRT_symposium_or_Conference', 0, 1, 21.0]}"

]

},

"execution_count": 20, "metadata": {},

"output_type": "execute_result"

}

],

"source": [ "dict_total_act"

]

},

{

"cell_type": "code", "execution_count": 21, "metadata": {}, "outputs": [

{

"data": {

"text/html": [ "<div>\n",

"<style scoped>\n",

" .dataframe tbody tr th:only-of-type {\n", " vertical-align: middle;\n",

" }\n",

"\n",

" .dataframe tbody tr th {\n", " vertical-align: top;\n",

" }\n",

"\n",

" .dataframe thead th {\n", " text-align: right;\n",

" }\n", "</style>\n",

"<table border=\"1\" class=\"dataframe\">\n", " <thead>\n",

" <tr style=\"text-align: right;\">\n", " <th></th>\n",

" <th>ActDes</th>\n",

" <th>num_freq_unk</th>\n", " <th>num_len_unk</th>\n",

" <th>total_time (hr/year)</th>\n", " </tr>\n",

" </thead>\n", " <tbody>\n", " <tr>\n",

" <th>1</th>\n",

" <td>Meeting</td>\n", " <td>6</td>\n",

" <td>3</td>\n",

" <td>351.1</td>\n", " </tr>\n",

" <tr>\n",

" <th>2</th>\n",

" <td>Workshop/panel/boot_camp</td>\n", " <td>9</td>\n",

" <td>2</td>\n",

" <td>276.2</td>\n", " </tr>\n",

" <tr>\n",

" <th>3</th>\n",

" <td>Course</td>\n", " <td>0</td>\n",

" <td>3</td>\n",

" <td>1155.75</td>\n", " </tr>\n",

" <tr>\n",

" <th>4</th>\n",

" <td>NRT_seminar</td>\n", " <td>4</td>\n",

" <td>1</td>\n",

" <td>199.25</td>\n",

" </tr>\n",

" <tr>\n",

" <th>5</th>\n",

" <td>NRT_symposium_or_Conference</td>\n", " <td>0</td>\n",

" <td>1</td>\n",

" <td>21.0</td>\n", " </tr>\n",

" <tr>\n",

" <th>6</th>\n",

" <td>Retreat/social_activities</td>\n", " <td>1</td>\n",

" <td>1</td>\n",

" <td>25.083333</td>\n", " </tr>\n",

" <tr>\n",

" <th>7</th>\n",

" <td>Internship</td>\n", " <td>1</td>\n",

" <td>1</td>\n", " <td>0</td>\n", " </tr>\n",

" <tr>\n",

" <th>8</th>\n",

" <td>Outreach_activity</td>\n", " <td>1</td>\n",

" <td>1</td>\n",

" <td>196.908333</td>\n",

" </tr>\n",

" <tr>\n",

" <th>9</th>\n",

" <td>Other</td>\n", " <td>9</td>\n",

" <td>5</td>\n",

" <td>179.258333</td>\n",

" </tr>\n",

" <tr>\n",

" <th>88</th>\n",

" <td>Unknown</td>\n", " <td>2</td>\n",

" <td>0</td>\n",

" <td>62.0</td>\n", " </tr>\n",

" </tbody>\n", "</table>\n", "</div>"

],

"text/plain": [

" ActDes num_freq_unk num_len_unk total_time (hr/year)\n",

"1 Meeting 6 3 351.1\n",

"2 Workshop/panel/boot_camp 9 2 276.2\n",

"3 Course 0 3 1155.75\n",

"4 NRT_seminar 4 1 199.25\n",

"5 NRT_symposium_or_Conference 0 1 21.0\n",

| "6 | Retreat/social_activities |  |  | 1 |  |  | 1 | 25.083333\n", |
| --- | --- | --- | --- | --- | --- | --- | --- | --- |
| "7 | Internship |  | 1 |  |  | 1 |  | 0\n", |
| "8 | Outreach_activity |  |  | 1 |  |  | 1 | 196.908333\n", |
| "9 | Other | 9 |  |  | 5 |  |  | 179.258333\n", |
| "88 | Unknown |  |  | 2 |  |  | 0 | 62.0" |
| ] |  |  |  |  |  |  |  |  |

},

"execution_count": 21, "metadata": {},

"output_type": "execute_result"

}

],

"source": [ "df_total_act"

]

},

{

"cell_type": "markdown", "metadata": {},

"source": [

"### Activity time per institution"

]

},

{

"cell_type": "code", "execution_count": 22, "metadata": {},

"outputs": [], "source": [

"dict_total_act_per_inst, df_total_act_per_inst = \\\n", " time_spent_per_inst(data, cat_dict, \"ActDes\")"

]

},

{

"cell_type": "code", "execution_count": 23, "metadata": {}, "outputs": [

{

"data": {

"text/html": [ "<div>\n",

"<style scoped>\n",

" .dataframe tbody tr th:only-of-type {\n", " vertical-align: middle;\n",

" }\n",

"\n",

" .dataframe tbody tr th {\n", " vertical-align: top;\n",

" }\n",

"\n",

" .dataframe thead th {\n", " text-align: right;\n",

" }\n",

"</style>\n",

"<table border=\"1\" class=\"dataframe\">\n", " <thead>\n",

" <tr style=\"text-align: right;\">\n", " <th></th>\n",

" <th>1</th>\n", " <th>2</th>\n", " <th>3</th>\n", " <th>4</th>\n", " <th>5</th>\n", " <th>6</th>\n", " <th>7</th>\n", " <th>8</th>\n", " <th>9</th>\n", " <th>88</th>\n", " </tr>\n",

" </thead>\n", " <tbody>\n", " <tr>\n",

" <th>1</th>\n",

" <td>9.0</td>\n", " <td>32.0</td>\n", " <td>60.0</td>\n", " <td>0.0</td>\n", " <td>0.0</td>\n", " <td>17.0</td>\n", " <td>0.0</td>\n", " <td>0.0</td>\n", " <td>0.0</td>\n", " <td>0.0</td>\n", " </tr>\n",

" <tr>\n",

" <th>2</th>\n",

" <td>138.5</td>\n", " <td>81.0</td>\n", " <td>160.0</td>\n", " <td>24.5</td>\n", " <td>12.0</td>\n", " <td>7.5</td>\n",

" <td>0.0</td>\n", " <td>24.5</td>\n", " <td>32.7</td>\n", " <td>0.0</td>\n", " </tr>\n",

" <tr>\n",

" <th>3</th>\n",

" <td>2.5</td>\n", " <td>15.0</td>\n", " <td>0.0</td>\n", " <td>0.0</td>\n", " <td>2.0</td>\n", " <td>0.0</td>\n", " <td>0.0</td>\n", " <td>3.5</td>\n",

" <td>5.0</td>\n", " <td>2.5</td>\n", " </tr>\n",

" <tr>\n",

" <th>4</th>\n",

" <td>2.0</td>\n", " <td>8.0</td>\n",

" <td>225.0</td>\n", " <td>1.2</td>\n",

" <td>0.0</td>\n", " <td>0.0</td>\n", " <td>0.0</td>\n", " <td>8.0</td>\n", " <td>8.0</td>\n", " <td>0.0</td>\n", " </tr>\n",

" <tr>\n",

" <th>5</th>\n",

" <td>0.0</td>\n", " <td>10.5</td>\n", " <td>0.0</td>\n", " <td>0.0</td>\n", " <td>0.0</td>\n", " <td>0.6</td>\n", " <td>0.0</td>\n", " <td>0.0</td>\n", " <td>0.0</td>\n", " <td>0.0</td>\n", " </tr>\n",

" <tr>\n",

" <th>6</th>\n",

" <td>2.5</td>\n", " <td>2.0</td>\n",

" <td>208.5</td>\n", " <td>0.0</td>\n",

" <td>0.0</td>\n", " <td>0.0</td>\n", " <td>0.0</td>\n", " <td>0.0</td>\n", " <td>0.0</td>\n", " <td>0.0</td>\n", " </tr>\n",

" <tr>\n",

" <th>7</th>\n",

" <td>9.5</td>\n", " <td>9.5</td>\n", " <td>0.0</td>\n", " <td>0.0</td>\n", " <td>0.0</td>\n", " <td>0.0</td>\n", " <td>0.0</td>\n", " <td>0.0</td>\n", " <td>0.0</td>\n", " <td>0.0</td>\n",

" </tr>\n",

" <tr>\n",

" <th>8</th>\n",

" <td>0.0</td>\n", " <td>0.0</td>\n", " <td>0.0</td>\n", " <td>0.0</td>\n", " <td>0.0</td>\n", " <td>0.0</td>\n", " <td>0.0</td>\n", " <td>75.9</td>\n", " <td>81.4</td>\n", " <td>45.0</td>\n", " </tr>\n",

" <tr>\n",

" <th>9</th>\n",

" <td>0.0</td>\n", " <td>1.5</td>\n", " <td>0.0</td>\n", " <td>18.0</td>\n", " <td>0.0</td>\n", " <td>0.0</td>\n", " <td>0.0</td>\n", " <td>0.0</td>\n", " <td>0.0</td>\n", " <td>4.5</td>\n", " </tr>\n",

" <tr>\n",

" <th>10</th>\n", " <td>0.0</td>\n", " <td>1.0</td>\n", " <td>0.0</td>\n",

" <td>24.0</td>\n", " <td>0.0</td>\n", " <td>0.0</td>\n", " <td>0.0</td>\n", " <td>0.0</td>\n", " <td>0.0</td>\n", " <td>0.0</td>\n", " </tr>\n",

" <tr>\n",

" <th>11</th>\n", " <td>2.0</td>\n", " <td>3.0</td>\n", " <td>0.0</td>\n", " <td>6.0</td>\n", " <td>0.0</td>\n", " <td>0.0</td>\n", " <td>0.0</td>\n", " <td>0.0</td>\n", " <td>1.0</td>\n", " <td>0.0</td>\n", " </tr>\n",

" <tr>\n",

" <th>12</th>\n", " <td>5.0</td>\n", " <td>5.0</td>\n",

" <td>90.0</td>\n", " <td>18.0</td>\n", " <td>0.0</td>\n", " <td>0.0</td>\n", " <td>0.0</td>\n", " <td>0.0</td>\n", " <td>0.0</td>\n", " <td>10.0</td>\n", " </tr>\n",

" <tr>\n",

" <th>13</th>\n", " <td>0.0</td>\n", " <td>0.0</td>\n",

" <td>26.0</td>\n", " <td>0.0</td>\n", " <td>0.0</td>\n", " <td>0.0</td>\n", " <td>0.0</td>\n", " <td>0.0</td>\n", " <td>0.0</td>\n", " <td>0.0</td>\n", " </tr>\n",

" <tr>\n",

" <th>14</th>\n",

" <td>18.0</td>\n", " <td>25.2</td>\n", " <td>22.5</td>\n", " <td>22.5</td>\n", " <td>0.0</td>\n", " <td>0.0</td>\n", " <td>0.0</td>\n", " <td>0.0</td>\n", " <td>0.0</td>\n", " <td>0.0</td>\n", " </tr>\n",

" <tr>\n",

" <th>15</th>\n",

" <td>75.6</td>\n", " <td>14.5</td>\n", " <td>67.5</td>\n", " <td>0.0</td>\n", " <td>0.0</td>\n", " <td>0.0</td>\n", " <td>0.0</td>\n", " <td>0.0</td>\n", " <td>16.6</td>\n", " <td>0.0</td>\n", " </tr>\n",

" <tr>\n",

" <th>16</th>\n",

" <td>22.5</td>\n",

" <td>24.0</td>\n", " <td>0.0</td>\n", " <td>0.0</td>\n", " <td>3.0</td>\n", " <td>0.0</td>\n", " <td>0.0</td>\n", " <td>0.0</td>\n", " <td>0.0</td>\n", " <td>0.0</td>\n", " </tr>\n",

" <tr>\n",

" <th>17</th>\n", " <td>0.0</td>\n", " <td>0.0</td>\n",

" <td>82.5</td>\n", " <td>0.0</td>\n", " <td>0.0</td>\n", " <td>0.0</td>\n", " <td>0.0</td>\n", " <td>0.0</td>\n", " <td>0.0</td>\n", " <td>0.0</td>\n", " </tr>\n",

" <tr>\n",

" <th>18</th>\n",

" <td>41.5</td>\n", " <td>0.0</td>\n",

" <td>157.5</td>\n", " <td>45.0</td>\n", " <td>0.0</td>\n",

" <td>0.0</td>\n", " <td>0.0</td>\n", " <td>72.0</td>\n", " <td>0.0</td>\n", " <td>0.0</td>\n", " </tr>\n",

" <tr>\n",

" <th>19</th>\n", " <td>0.0</td>\n",

" <td>27.0</td>\n", " <td>56.2</td>\n", " <td>0.0</td>\n", " <td>4.0</td>\n", " <td>0.0</td>\n", " <td>0.0</td>\n", " <td>3.0</td>\n", " <td>0.0</td>\n", " <td>0.0</td>\n", " </tr>\n",

" <tr>\n",

" <th>20</th>\n",

" <td>22.5</td>\n", " <td>17.0</td>\n", " <td>0.0</td>\n",

" <td>40.0</td>\n", " <td>0.0</td>\n", " <td>0.0</td>\n", " <td>0.0</td>\n", " <td>10.0</td>\n", " <td>34.5</td>\n", " <td>0.0</td>\n", " </tr>\n",

" </tbody>\n", "</table>\n", "</div>"

],

"text/plain": [

" 1 2 3 4 5 6 7 8 9 88\n",

"1 9.0 32.0 60.0 0.0 0.0 17.0 0.0 0.0 0.0 0.0\n",

"2 138.5 81.0 160.0 24.5 12.0 7.5 0.0 24.5 32.7 0.0\n",

| "3 | 2.5 15.0 0.0 0.0 2.0 0.0 0.0 3.5 5.0 2.5\n", | | |
| --- | --- | --- | --- |
| "4 | 2.0 8.0 225.0 1.2 0.0 0.0 0.0 8.0 8.0 0.0\n", | | |
| "5 | 0.0 10.5 0.0 0.0 0.0 0.6 0.0 0.0 0.0 0.0\n", | | |
| "6 | 2.5 2.0 208.5 0.0 0.0 0.0 0.0 0.0 0.0 0.0\n", | | |
| "7 | 9.5 9.5 0.0 0.0 0.0 0.0 0.0 0.0 0.0 0.0\n", | | |
| "8 | 0.0 0.0 0.0 0.0 0.0 0.0 0.0 75.9 81.4 45.0\n", | | |
| "9 | 0.0 1.5 0.0 18.0 0.0 0.0 0.0 0.0 0.0 4.5\n", | | |
| "10 | 0.0 1.0 0.0 24.0 0.0 0.0 0.0 0.0 0.0 0.0\n", | | |
| "11 | 2.0 3.0 0.0 6.0 0.0 0.0 0.0 0.0 1.0 0.0\n", | | |
| "12 | 5.0 5.0 90.0 18.0 0.0 0.0 0.0 0.0 0.0 10.0\n", | | |
| "13 | 0.0 0.0 26.0 0.0 0.0 0.0 0.0 0.0 0.0 0.0\n", | | |
| "14 | 18.0 25.2 22.5 22.5 0.0 0.0 0.0 0.0 0.0 0.0\n", | | |
| "15 | 75.6 14.5 67.5 0.0 0.0 0.0 0.0 0.0 16.6 0.0\n", | | |
| "16 | 22.5 24.0 0.0 0.0 3.0 0.0 0.0 0.0 0.0 0.0\n", | | |
| "17 | 0.0 0.0 82.5 0.0 0.0 0.0 0.0 0.0 0.0 0.0\n", | | |
| "18 | 41.5 0.0 157.5 45.0 0.0 0.0 0.0 72.0 0.0 0.0\n", | | |
| "19 | 0.0 27.0 56.2 0.0 4.0 | 0.0 0.0 | 3.0 0.0 0.0\n", |
| "20  ] | 22.5 17.0 0.0 40.0 0.0 | 0.0 0.0 | 10.0 34.5 0.0" |

},

"execution_count": 23, "metadata": {},

"output_type": "execute_result"

}

],

"source": [ "df_total_act_per_inst"

]

},

{

"cell_type": "markdown", "metadata": {},

"source": [

"### Number of individual total"

]

},

{

"cell_type": "code",

"execution_count": 24, "metadata": {},

"outputs": [], "source": [

"_, df_indiv_act = get_num_indiv(data, cat_dict, \"ActDes\")"

]

},

{

"cell_type": "code", "execution_count": 25, "metadata": {}, "outputs": [

{

"data": {

"text/html": [ "<div>\n",

"<style scoped>\n",

" .dataframe tbody tr th:only-of-type {\n", " vertical-align: middle;\n",

" }\n",

"\n",

" .dataframe tbody tr th {\n", " vertical-align: top;\n",

" }\n",

"\n",

" .dataframe thead th {\n", " text-align: right;\n",

" }\n", "</style>\n",

"<table border=\"1\" class=\"dataframe\">\n", " <thead>\n",

" <tr style=\"text-align: right;\">\n", " <th></th>\n",

" <th>ActDes</th>\n",

" <th>TNumPart</th>\n", " <th>NumFun</th>\n",

" <th>NumNFun</th>\n", " <th>NumNTrain</th>\n", " </tr>\n",

" </thead>\n", " <tbody>\n", " <tr>\n",

" <th>1</th>\n",

" <td>Meeting</td>\n", " <td>969</td>\n",

" <td>184</td>\n", " <td>117</td>\n", " <td>212</td>\n", " </tr>\n",

" <tr>\n",

" <th>2</th>\n",

" <td>Workshop/panel/boot_camp</td>\n", " <td>1646</td>\n",

" <td>313</td>\n",

" <td>226</td>\n", " <td>253</td>\n", " </tr>\n",

" <tr>\n",

" <th>3</th>\n",

" <td>Course</td>\n", " <td>977</td>\n",

" <td>230</td>\n", " <td>183</td>\n", " <td>326</td>\n", " </tr>\n",

" <tr>\n",

" <th>4</th>\n",

" <td>NRT_seminar</td>\n", " <td>936</td>\n",

" <td>352</td>\n", " <td>265</td>\n", " <td>221</td>\n", " </tr>\n",

" <tr>\n",

" <th>5</th>\n",

" <td>NRT_symposium_or_Conference</td>\n", " <td>363</td>\n",

" <td>32</td>\n", " <td>22</td>\n", " <td>16</td>\n", " </tr>\n",

" <tr>\n",

" <th>6</th>\n",

" <td>Retreat/social_activities</td>\n", " <td>77</td>\n",

" <td>41</td>\n", " <td>17</td>\n", " <td>2</td>\n", " </tr>\n",

" <tr>\n",

" <th>7</th>\n",

" <td>Internship</td>\n", " <td>4</td>\n",

" <td>1</td>\n", " <td>3</td>\n", " <td>0</td>\n", " </tr>\n",

" <tr>\n",

" <th>8</th>\n",

" <td>Outreach_activity</td>\n", " <td>1062</td>\n",

" <td>74</td>\n", " <td>91</td>\n", " <td>426</td>\n", " </tr>\n",

" <tr>\n",

" <th>9</th>\n",

" <td>Other</td>\n",

" <td>2215</td>\n", " <td>122</td>\n", " <td>136</td>\n", " <td>1251</td>\n", " </tr>\n",

" <tr>\n",

" <th>88</th>\n",

" <td>Unknown</td>\n", " <td>1190</td>\n",

" <td>32</td>\n", " <td>35</td>\n", " <td>148</td>\n", " </tr>\n",

" </tbody>\n", "</table>\n", "</div>"

],

"text/plain": [

" ActDes TNumPart NumFun NumNFun NumNTrain\n",

"1 Meeting 969 184 117 212\n",

"2 Workshop/panel/boot_camp 1646 313 226 253\n",

"3 Course 977 230 183 326\n",

"4 NRT_seminar 936 352 265 221\n",

"5 NRT_symposium_or_Conference 363 32 22 16\n",

| "6 | Retreat/social_activities | 77 | 41 | 17 | | 2\n", |
| --- | --- | --- | --- | --- | --- | --- |
| "7 | Internship 4 | 1 | 3 | 0\n", | |  |
| "8 | Outreach_activity | 1062 | 74 | 91 | | 426\n", |
| "9 | Other 2215 122 | | 136 | | 1251\n", | |
| "88  ]  }, | Unknown 1190 | | 32 35 | | 148" | |

"execution_count": 25, "metadata": {},

"output_type": "execute_result"

}

],

"source": [ "df_indiv_act"

]

},

{

"cell_type": "markdown", "metadata": {},

"source": [

"### Number of individual per institution"

]

},

{

"cell_type": "code", "execution_count": 26, "metadata": {},

"outputs": [], "source": [

"df_ntp_act_inst, df_nf_act_inst, df_nnf_act_inst, df_nnt_act_inst =\\\n",

" get_num_indiv_per_inst(data, cat_dict, \"ActDes\")"

]

},

{

"cell_type": "code", "execution_count": 27, "metadata": {}, "outputs": [

{

"data": {

"text/html": [ "<div>\n",

"<style scoped>\n",

" .dataframe tbody tr th:only-of-type {\n", " vertical-align: middle;\n",

" }\n",

"\n",

" .dataframe tbody tr th {\n", " vertical-align: top;\n",

" }\n",

"\n",

" .dataframe thead th {\n", " text-align: right;\n",

" }\n", "</style>\n",

"<table border=\"1\" class=\"dataframe\">\n", " <thead>\n",

" <tr style=\"text-align: right;\">\n", " <th></th>\n",

" <th>1</th>\n", " <th>2</th>\n", " <th>3</th>\n", " <th>4</th>\n", " <th>5</th>\n", " <th>6</th>\n", " <th>7</th>\n", " <th>8</th>\n", " <th>9</th>\n", " <th>88</th>\n", " </tr>\n",

" </thead>\n", " <tbody>\n", " <tr>\n",

" <th>1</th>\n", " <td>42</td>\n", " <td>38</td>\n", " <td>69</td>\n", " <td>0</td>\n", " <td>0</td>\n", " <td>24</td>\n", " <td>0</td>\n", " <td>0</td>\n", " <td>0</td>\n", " <td>0</td>\n",

" </tr>\n",

" <tr>\n",

" <th>2</th>\n",

" <td>166</td>\n", " <td>139</td>\n", " <td>85</td>\n", " <td>96</td>\n", " <td>60</td>\n", " <td>10</td>\n", " <td>0</td>\n",

" <td>746</td>\n", " <td>146</td>\n", " <td>0</td>\n",

" </tr>\n",

" <tr>\n",

" <th>3</th>\n", " <td>16</td>\n",

" <td>185</td>\n", " <td>0</td>\n",

" <td>0</td>\n", " <td>77</td>\n", " <td>0</td>\n", " <td>0</td>\n", " <td>37</td>\n", " <td>79</td>\n", " <td>16</td>\n", " </tr>\n",

" <tr>\n",

" <th>4</th>\n", " <td>23</td>\n", " <td>0</td>\n", " <td>89</td>\n", " <td>19</td>\n", " <td>0</td>\n", " <td>0</td>\n", " <td>0</td>\n", " <td>13</td>\n",

" <td>188</td>\n", " <td>0</td>\n",

" </tr>\n",

" <tr>\n",

" <th>5</th>\n", " <td>0</td>\n",

" <td>132</td>\n", " <td>0</td>\n",

" <td>0</td>\n", " <td>0</td>\n", " <td>32</td>\n", " <td>0</td>\n", " <td>0</td>\n", " <td>0</td>\n", " <td>0</td>\n", " </tr>\n",

" <tr>\n",

" <th>6</th>\n", " <td>22</td>\n", " <td>8</td>\n", " <td>36</td>\n", " <td>0</td>\n", " <td>0</td>\n", " <td>0</td>\n", " <td>0</td>\n", " <td>0</td>\n", " <td>0</td>\n", " <td>0</td>\n", " </tr>\n",

" <tr>\n",

" <th>7</th>\n",

" <td>150</td>\n", " <td>150</td>\n", " <td>0</td>\n",

" <td>0</td>\n", " <td>0</td>\n", " <td>0</td>\n", " <td>0</td>\n", " <td>0</td>\n", " <td>0</td>\n", " <td>0</td>\n", " </tr>\n",

" <tr>\n",

" <th>8</th>\n", " <td>0</td>\n", " <td>0</td>\n", " <td>0</td>\n", " <td>0</td>\n", " <td>0</td>\n", " <td>0</td>\n", " <td>0</td>\n",

" <td>235</td>\n", " <td>472</td>\n", " <td>175</td>\n", " </tr>\n",

" <tr>\n",

" <th>9</th>\n", " <td>0</td>\n", " <td>7</td>\n", " <td>0</td>\n", " <td>3</td>\n", " <td>0</td>\n", " <td>0</td>\n", " <td>0</td>\n", " <td>0</td>\n", " <td>0</td>\n", " <td>24</td>\n", " </tr>\n",

" <tr>\n",

" <th>10</th>\n", " <td>0</td>\n",

" <td>13</td>\n", " <td>0</td>\n",

" <td>406</td>\n", " <td>0</td>\n",

" <td>0</td>\n", " <td>0</td>\n", " <td>0</td>\n", " <td>0</td>\n", " <td>0</td>\n", " </tr>\n",

" <tr>\n",

" <th>11</th>\n", " <td>12</td>\n", " <td>57</td>\n", " <td>0</td>\n",

" <td>172</td>\n", " <td>0</td>\n",

" <td>0</td>\n", " <td>0</td>\n", " <td>0</td>\n", " <td>6</td>\n", " <td>0</td>\n", " </tr>\n",

" <tr>\n",

" <th>12</th>\n", " <td>169</td>\n", " <td>552</td>\n", " <td>7</td>\n",

" <td>24</td>\n", " <td>100</td>\n", " <td>0</td>\n",

" <td>0</td>\n", " <td>0</td>\n", " <td>0</td>\n",

" <td>975</td>\n", " </tr>\n",

" <tr>\n",

" <th>13</th>\n", " <td>0</td>\n", " <td>0</td>\n",

" <td>440</td>\n", " <td>0</td>\n",

" <td>0</td>\n", " <td>0</td>\n", " <td>0</td>\n", " <td>0</td>\n", " <td>0</td>\n", " <td>0</td>\n", " </tr>\n",

" <tr>\n",

" <th>14</th>\n", " <td>99</td>\n", " <td>124</td>\n", " <td>8</td>\n",

" <td>99</td>\n", " <td>0</td>\n", " <td>0</td>\n", " <td>0</td>\n", " <td>0</td>\n", " <td>0</td>\n", " <td>0</td>\n", " </tr>\n",

" <tr>\n",

" <th>15</th>\n", " <td>93</td>\n", " <td>102</td>\n", " <td>15</td>\n", " <td>0</td>\n",

" <td>0</td>\n", " <td>0</td>\n", " <td>0</td>\n", " <td>0</td>\n", " <td>13</td>\n", " <td>0</td>\n", " </tr>\n",

" <tr>\n",

" <th>16</th>\n", " <td>39</td>\n", " <td>48</td>\n", " <td>76</td>\n", " <td>3</td>\n", " <td>81</td>\n", " <td>0</td>\n", " <td>0</td>\n", " <td>0</td>\n", " <td>96</td>\n", " <td>0</td>\n", " </tr>\n",

" <tr>\n",

" <th>17</th>\n", " <td>0</td>\n", " <td>0</td>\n", " <td>50</td>\n", " <td>0</td>\n", " <td>0</td>\n", " <td>0</td>\n", " <td>0</td>\n", " <td>0</td>\n", " <td>0</td>\n", " <td>0</td>\n", " </tr>\n",

" <tr>\n",

" <th>18</th>\n", " <td>100</td>\n", " <td>0</td>\n",

" <td>91</td>\n", " <td>51</td>\n", " <td>0</td>\n",

" <td>0</td>\n", " <td>0</td>\n", " <td>10</td>\n", " <td>0</td>\n", " <td>0</td>\n", " </tr>\n",

" <tr>\n",

" <th>19</th>\n", " <td>0</td>\n", " <td>22</td>\n", " <td>11</td>\n", " <td>0</td>\n", " <td>45</td>\n", " <td>0</td>\n", " <td>0</td>\n", " <td>6</td>\n", " <td>0</td>\n", " <td>0</td>\n", " </tr>\n",

" <tr>\n",

" <th>20</th>\n", " <td>38</td>\n", " <td>69</td>\n", " <td>0</td>\n", " <td>63</td>\n", " <td>0</td>\n", " <td>11</td>\n", " <td>4</td>\n", " <td>15</td>\n",

" <td>1215</td>\n", " <td>0</td>\n",

" </tr>\n",

" </tbody>\n", "</table>\n", "</div>"

],

"text/plain": [

| " | 1 2 3 4 | | | 5 6 | | 7 8 | 9 88\n", | | |
| --- | --- | --- | --- | --- | --- | --- | --- | --- | --- |
| "1 | 42 38 69 | | | 0 0 | | 24 0 | 0 0 0\n", | | |
| "2 | 166 139 85 | | | 96 60 10 | | | 0 746 146 0\n", | | |
| "3 | 16 185 0 0 | | | | 77 0 0 | | 37 | 79 16\n", | |
| "4 | 23 0 89 19 | | | | 0 0 0 | | 13 | 188 0\n", | |
| "5 | 0 132 0 0 | | | | 0 32 0 | | 0 | 0 0\n", | |
| "6 | 22 8 36 0 | | | | 0 0 0 | | 0 | 0 0\n", | |
| "7 | 150 150 0 0 | | | | 0 0 0 | | 0 | 0 0\n", | |
| "8 | 0 | 0 0 0 0 | | | 0 0 235 472 | | | | 175\n", |
| "9 | 0 | 7 0 3 0 | | | 0 0 0 0 24\n", | | | | |
| "10 | 0 | 13 0 406 | | | 0 0 0 0 0 0\n", | | | | |
| "11 | 12 | 57 0 172 | | | 0 0 0 0 6 0\n", | | | | |
| "12 | 169 | 552 7 | | 24 | 100 0 0 | | | 0 0 975\n", | |
| "13 | 0 | 0 440 | | 0 | 0 0 0 0 | | | 0 0\n", | |
| "14 | 99 124 | | 8 | 99 | 0 0 0 0 | | | 0 0\n", | |
| "15 | 93 102 | | 15 | 0 | 0 0 0 0 | | | 13 0\n", | |
| "16 | 39 48 | | 76 | 3 | 81 0 0 0 | | | 96 0\n", | |

"17 0 0 50 0 0 0 0 0 0 0\n",

"18 100 0 91 51 0 0 0 10 0 0\n",

"19 0 22 11 0 45 0 0 6 0 0\n",

"20 38 69 0 63 0 11 4 15 1215 0"

]

},

"execution_count": 27, "metadata": {},

"output_type": "execute_result"

}

],

"source": [ "df_ntp_act_inst"

]

},

{

"cell_type": "markdown", "metadata": {},

"source": [

"## Analysis: prof skills "

]

},

{

"cell_type": "markdown", "metadata": {},

"source": [

"### Get ID-prof skill counts"

]

},

{

"cell_type": "code", "execution_count": 28, "metadata": {},

"outputs": [], "source": [

"# Count unique ID-ProfSkill combinations\n",

"_, df_id_prof = joint_two_columns(data, cat_dict, \"ID\", \"ProfSkill\", 1)"

]

},

{

"cell_type": "markdown", "metadata": {},

"source": [

"### ProfSkill time decicated"

]

},

{

"cell_type": "code", "execution_count": 29, "metadata": {},

"outputs": [], "source": [

"_, df_total_prof = time_spent(data, cat_dict, \"ProfSkill\")\n",

"_, df_total_prof_per_inst = time_spent_per_inst(data, cat_dict, \"ProfSkill\")"

]

},

{

"cell_type": "markdown", "metadata": {},

"source": [

"### Number of individuals involved"

]

},

{

"cell_type": "code", "execution_count": 30, "metadata": {},

"outputs": [], "source": [

"_, df_indiv_prof = get_num_indiv(data, cat_dict, \"ProfSkill\")\n", "df_ntp_prof_inst, df_nf_prof_inst, df_nnf_prof_inst, df_nnt_prof_inst =\\\n", " get_num_indiv_per_inst(data, cat_dict, \"ProfSkill\")"

]

},

{

"cell_type": "markdown", "metadata": {},

"source": [

"## Analysis: stakeholder "

]

},

{

"cell_type": "markdown", "metadata": {},

"source": [

"### Get ID-StkSrvd counts"

]

},

{

"cell_type": "code", "execution_count": 31, "metadata": {},

"outputs": [], "source": [

"# Count unique ID-ActDes combinations\n",

"_, df_id_prof_stk = joint_two_columns(data, cat_dict, \"ID\", \"StkSrvd\", 1)"

]

},

{

"cell_type": "markdown", "metadata": {},

"source": [

"### Stakeholder time decicated"

]

},

{

"cell_type": "code", "execution_count": 32,

"metadata": {},

"outputs": [], "source": [

"_, df_total_stk = time_spent(data, cat_dict, \"StkSrvd\")\n",

"_, df_total_stk_per_inst = time_spent_per_inst(data, cat_dict, \"StkSrvd\")"

]

},

{

"cell_type": "markdown", "metadata": {},

"source": [

"### Number of individuals involved"

]

},

{

"cell_type": "code", "execution_count": 33, "metadata": {},

"outputs": [], "source": [

"_, df_indiv_stk = get_num_indiv(data, cat_dict, \"StkSrvd\")\n", "df_ntp_stk_inst, df_nf_stk_inst, df_nnf_stk_inst, df_nnt_stk_inst =\\\n", " get_num_indiv_per_inst(data, cat_dict, \"StkSrvd\")"

]

},

{

"cell_type": "markdown", "metadata": {},

"source": [

"## Pairwise for ActDes, ProfSkil, and StkSrvd "

]

},

{

"cell_type": "markdown", "metadata": {},

"source": [

"### ActDes vs. ProfSkill"

]

},

{

"cell_type": "code", "execution_count": 34, "metadata": {},

"outputs": [], "source": [

"_, df_act_prof = joint_two_columns(data, cat_dict, \"ActDes\", \"ProfSkill\", \n", " col1_is_iid=0)"

]

},

{

"cell_type": "code", "execution_count": 35, "metadata": {}, "outputs": [

{

"data": {

"text/html": [ "<div>\n",

"<style scoped>\n",

" .dataframe tbody tr th:only-of-type {\n", " vertical-align: middle;\n",

" }\n",

"\n",

" .dataframe tbody tr th {\n", " vertical-align: top;\n",

" }\n",

"\n",

" .dataframe thead th {\n", " text-align: right;\n",

" }\n", "</style>\n",

"<table border=\"1\" class=\"dataframe\">\n", " <thead>\n",

" <tr style=\"text-align: right;\">\n", " <th></th>\n",

" <th>1_Training_in_interdisciplinary_skills</th>\n", " <th>2_Training_in_disciplinary_skills</th>\n",

" <th>3_Critical_thinking</th>\n",

" <th>4_Critical_thinking-ethics</th>\n",

" <th>5_Team_science_&amp;_collaboration_skill</th>\n", " <th>6_Teaching_&amp;_Mentoring_</th>\n",

" <th>7_Entrepreneurship_and_IP</th>\n",

" <th>8_Communication_-_academic</th>\n",

" <th>9_Communication_-_non-academic</th>\n", " <th>10_DEI/Broadening_participation</th>\n",

" <th>11_Job_readiness</th>\n", " <th>12_Others</th>\n",

" <th>13_Outreach</th>\n", " <th>88_Unknown</th>\n", " </tr>\n",

" </thead>\n", " <tbody>\n", " <tr>\n",

" <th>1_Meeting</th>\n", " <td>10</td>\n",

" <td>6</td>\n", " <td>2</td>\n", " <td>0</td>\n", " <td>20</td>\n", " <td>13</td>\n", " <td>1</td>\n", " <td>19</td>\n", " <td>11</td>\n", " <td>3</td>\n", " <td>14</td>\n", " <td>3</td>\n", " <td>2</td>\n", " <td>4</td>\n",

" </tr>\n",

" <tr>\n",

" <th>2_Workshop/panel/boot_camp</th>\n", " <td>11</td>\n",

" <td>8</td>\n", " <td>9</td>\n", " <td>1</td>\n", " <td>10</td>\n", " <td>16</td>\n", " <td>6</td>\n", " <td>23</td>\n", " <td>16</td>\n", " <td>9</td>\n", " <td>24</td>\n", " <td>18</td>\n", " <td>3</td>\n", " <td>3</td>\n", " </tr>\n",

" <tr>\n",

" <th>3_Course</th>\n", " <td>15</td>\n",

" <td>14</td>\n", " <td>8</td>\n", " <td>5</td>\n", " <td>17</td>\n", " <td>2</td>\n", " <td>0</td>\n", " <td>17</td>\n", " <td>13</td>\n", " <td>7</td>\n", " <td>19</td>\n", " <td>5</td>\n", " <td>0</td>\n", " <td>2</td>\n", " </tr>\n",

" <tr>\n",

" <th>4_NRT_seminar</th>\n", " <td>10</td>\n",

" <td>17</td>\n", " <td>1</td>\n", " <td>2</td>\n", " <td>3</td>\n", " <td>0</td>\n", " <td>0</td>\n", " <td>5</td>\n", " <td>4</td>\n", " <td>6</td>\n", " <td>12</td>\n", " <td>7</td>\n", " <td>0</td>\n", " <td>5</td>\n", " </tr>\n",

" <tr>\n",

" <th>5_NRT_symposium_or_Conference</th>\n",

" <td>2</td>\n", " <td>0</td>\n", " <td>1</td>\n", " <td>0</td>\n", " <td>3</td>\n", " <td>0</td>\n", " <td>0</td>\n", " <td>5</td>\n", " <td>0</td>\n", " <td>0</td>\n", " <td>2</td>\n", " <td>3</td>\n", " <td>0</td>\n", " <td>0</td>\n", " </tr>\n",

" <tr>\n",

" <th>6_Retreat/social_activities</th>\n", " <td>0</td>\n",

" <td>1</td>\n", " <td>0</td>\n", " <td>0</td>\n", " <td>3</td>\n", " <td>0</td>\n", " <td>0</td>\n", " <td>2</td>\n", " <td>0</td>\n", " <td>0</td>\n", " <td>3</td>\n", " <td>1</td>\n", " <td>0</td>\n", " <td>1</td>\n", " </tr>\n",

" <tr>\n",

" <th>7_Internship</th>\n", " <td>0</td>\n",

" <td>0</td>\n", " <td>0</td>\n", " <td>0</td>\n", " <td>0</td>\n", " <td>0</td>\n", " <td>0</td>\n", " <td>0</td>\n", " <td>0</td>\n", " <td>0</td>\n", " <td>1</td>\n", " <td>0</td>\n", " <td>0</td>\n", " <td>0</td>\n", " </tr>\n",

" <tr>\n",

" <th>8_Outreach_activity</th>\n", " <td>1</td>\n",

" <td>0</td>\n", " <td>3</td>\n",

" <td>0</td>\n", " <td>2</td>\n", " <td>2</td>\n", " <td>0</td>\n", " <td>10</td>\n", " <td>17</td>\n", " <td>0</td>\n", " <td>7</td>\n", " <td>2</td>\n", " <td>10</td>\n", " <td>0</td>\n", " </tr>\n",

" <tr>\n",

" <th>9_Other</th>\n", " <td>1</td>\n",

" <td>6</td>\n", " <td>4</td>\n", " <td>0</td>\n", " <td>3</td>\n", " <td>4</td>\n", " <td>0</td>\n", " <td>19</td>\n", " <td>13</td>\n", " <td>3</td>\n", " <td>13</td>\n", " <td>8</td>\n", " <td>5</td>\n", " <td>1</td>\n", " </tr>\n",

" <tr>\n",

" <th>88_Unknown</th>\n", " <td>0</td>\n",

" <td>0</td>\n", " <td>1</td>\n", " <td>0</td>\n", " <td>0</td>\n", " <td>0</td>\n", " <td>0</td>\n", " <td>2</td>\n", " <td>1</td>\n", " <td>0</td>\n", " <td>5</td>\n", " <td>3</td>\n", " <td>1</td>\n", " <td>1</td>\n", " </tr>\n",

" <tr>\n",

" <th>99_no_response_or_description</th>\n", " <td>0</td>\n",

" <td>0</td>\n", " <td>0</td>\n", " <td>0</td>\n", " <td>0</td>\n", " <td>0</td>\n",

" <td>0</td>\n", " <td>0</td>\n", " <td>0</td>\n", " <td>0</td>\n", " <td>0</td>\n", " <td>0</td>\n", " <td>0</td>\n", " <td>0</td>\n", " </tr>\n",

" </tbody>\n", "</table>\n", "</div>"

],

"text/plain": [

" 1_Training_in_interdisciplinary_skills \\\n", "1_Meeting 10 \n",

"2_Workshop/panel/boot_camp 11 \n",

"3_Course 15 \n",

"4_NRT_seminar 10 \n",

"5_NRT_symposium_or_Conference 2 \n",

"6_Retreat/social_activities 0 \n",

"7_Internship 0 \n",

"8_Outreach_activity 1 \n",

"9_Other 1 \n",

"88_Unknown 0 \n",

"99_no_response_or_description 0 \n", "\n",

" 2_Training_in_disciplinary_skills \\\n", "1_Meeting 6 \n",

"2_Workshop/panel/boot_camp 8 \n",

"3_Course 14 \n",

"4_NRT_seminar 17 \n",

"5_NRT_symposium_or_Conference 0 \n",

"6_Retreat/social_activities 1 \n",

"7_Internship 0 \n",

"8_Outreach_activity 0 \n",

"9_Other 6 \n",

"88_Unknown 0 \n",

"99_no_response_or_description 0 \n", "\n",

" 3_Critical_thinking \\\n", "1_Meeting 2 \n",

"2_Workshop/panel/boot_camp 9 \n",

"3_Course 8 \n",

"4_NRT_seminar 1 \n",

"5_NRT_symposium_or_Conference 1 \n",

"6_Retreat/social_activities 0 \n",

"7_Internship 0 \n",

"8_Outreach_activity 3 \n",

"9_Other 4 \n",

"88_Unknown 1 \n",

"99_no_response_or_description 0 \n", "\n",

" 4_Critical_thinking-ethics \\\n",

"1_Meeting 0 \n",

"2_Workshop/panel/boot_camp 1 \n",

"3_Course 5 \n",

"4_NRT_seminar 2 \n",

"5_NRT_symposium_or_Conference 0 \n",

"6_Retreat/social_activities 0 \n",

"7_Internship 0 \n",

"8_Outreach_activity 0 \n",

"9_Other 0 \n",

"88_Unknown 0 \n",

"99_no_response_or_description 0 \n", "\n",

" 5_Team_science_&_collaboration_skill \\\n", "1_Meeting 20 \n",

"2_Workshop/panel/boot_camp 10 \n",

"3_Course 17 \n",

"4_NRT_seminar 3 \n",

"5_NRT_symposium_or_Conference 3 \n",

"6_Retreat/social_activities 3 \n",

"7_Internship 0 \n",

"8_Outreach_activity 2 \n",

"9_Other 3 \n",

"88_Unknown 0 \n",

"99_no_response_or_description 0 \n", "\n",

" 6_Teaching_&_Mentoring_ \\\n", "1_Meeting 13 \n",

"2_Workshop/panel/boot_camp 16 \n",

"3_Course 2 \n",

"4_NRT_seminar 0 \n",

"5_NRT_symposium_or_Conference 0 \n",

"6_Retreat/social_activities 0 \n",

"7_Internship 0 \n",

"8_Outreach_activity 2 \n",

"9_Other 4 \n",

"88_Unknown 0 \n",

"99_no_response_or_description 0 \n", "\n",

" 7_Entrepreneurship_and_IP \\\n", "1_Meeting 1 \n",

"2_Workshop/panel/boot_camp 6 \n",

"3_Course 0 \n",

"4_NRT_seminar 0 \n",

"5_NRT_symposium_or_Conference 0 \n",

"6_Retreat/social_activities 0 \n",

"7_Internship 0 \n",

"8_Outreach_activity 0 \n",

"9_Other 0 \n",

"88_Unknown 0 \n",

"99_no_response_or_description 0 \n", "\n",

" 8_Communication_-_academic \\\n", "1_Meeting 19 \n",

"2_Workshop/panel/boot_camp 23 \n",

"3_Course 17 \n",

"4_NRT_seminar 5 \n",

"5_NRT_symposium_or_Conference 5 \n",

"6_Retreat/social_activities 2 \n",

"7_Internship 0 \n",

"8_Outreach_activity 10 \n",

"9_Other 19 \n",

"88_Unknown 2 \n",

"99_no_response_or_description 0 \n", "\n",

" 9_Communication_-_non-academic \\\n", "1_Meeting 11 \n",

"2_Workshop/panel/boot_camp 16 \n",

"3_Course 13 \n",

"4_NRT_seminar 4 \n",

"5_NRT_symposium_or_Conference 0 \n",

"6_Retreat/social_activities 0 \n",

"7_Internship 0 \n",

"8_Outreach_activity 17 \n",

"9_Other 13 \n",

"88_Unknown 1 \n",

"99_no_response_or_description 0 \n", "\n",

" 10_DEI/Broadening_participation \\\n", "1_Meeting 3 \n",

"2_Workshop/panel/boot_camp 9 \n",

"3_Course 7 \n",

"4_NRT_seminar 6 \n",

"5_NRT_symposium_or_Conference 0 \n",

"6_Retreat/social_activities 0 \n",

"7_Internship 0 \n",

"8_Outreach_activity 0 \n",

"9_Other 3 \n",

"88_Unknown 0 \n",

"99_no_response_or_description 0 \n", "\n",

" 11_Job_readiness 12_Others 13_Outreach \\\n", "1_Meeting 14 3 2 \n",

| 18 | 3 | \n", |
| --- | --- | --- |
| 0 \n",  0 | \n", |  |
| 3 |  | 0 \n", |
| 0 | \n", |  |

"2_Workshop/panel/boot_camp 24

"3_Course 19 5

"4_NRT_seminar 12 7

"5_NRT_symposium_or_Conference 2

"6_Retreat/social_activities 3 1

| "7_Internship "8_Outreach_activity  "9_Other | 1  13 | 7 | 0  8 | 2 | 0  5 | \n",  10 \n",  \n", |
| --- | --- | --- | --- | --- | --- | --- |
| "88_Unknown | 5 | | 3 |  | | 1 \n", |
| "99_no_response_or_description "\n", |  | | 0 | 0 | | 0 \n", |

" 88_Unknown \n", "1_Meeting 4 \n",

"2_Workshop/panel/boot_camp 3 \n",

"3_Course 2 \n",

"4_NRT_seminar 5 \n",

"5_NRT_symposium_or_Conference 0 \n",

"6_Retreat/social_activities 1 \n",

"7_Internship 0 \n",

"8_Outreach_activity 0 \n",

"9_Other 1 \n",

"88_Unknown 1 \n",

"99_no_response_or_description 0 "

]

},

"execution_count": 35, "metadata": {},

"output_type": "execute_result"

}

],

"source": [ "df_act_prof"

]

},

{

"cell_type": "markdown", "metadata": {},

"source": [

"### ActDes vs. StkSrvd"

]

},

{

"cell_type": "code", "execution_count": 36, "metadata": {},

"outputs": [], "source": [

"_, df_act_stk = joint_two_columns(data, cat_dict, \"ActDes\", \"StkSrvd\", \n", " col1_is_iid=0)"

]

},

{

"cell_type": "markdown", "metadata": {},

"source": [

"### ProfSkill vs. StkSrvd"

]

},

{

"cell_type": "code", "execution_count": 37, "metadata": {},

"outputs": [], "source": [

"_, df_prof_stk = joint_two_columns(data, cat_dict, \"ProfSkill\", \"StkSrvd\", \n", " col1_is_iid=0)"

]

},

{

"cell_type": "markdown",

"metadata": {}, "source": [

"## Output dataframes to excel "

]

},

{

"cell_type": "code", "execution_count": 38, "metadata": {},

"outputs": [], "source": [

"excel_data = work_dir / 'table_analysis_RAWDATA.xlsx'\n", "excel_act = work_dir / 'table_analysis_ActDes.xlsx'\n", "excel_prof = work_dir / 'table_analysis_ProfSkill.xlsx'\n", "excel_stk = work_dir / 'table_analysis_StkSrvd.xlsx'\n", "excel_pair = work_dir / 'table_analysis_pairwise.xlsx'"

]

},

{

"cell_type": "code", "execution_count": 39, "metadata": {},

"outputs": [], "source": [

"with pd.ExcelWriter(excel_data) as writer:\n", " # Original data\n",

" data.to_excel(writer, sheet_name=\"tiebreak combine 10-30\")\n", "\n",

"with pd.ExcelWriter(excel_act) as writer:\n", " # ActDes related\n",

" df_id_act.to_excel(writer, sheet_name=\"count-ID-ActDes\")\n", " df_total_act.to_excel(writer, sheet_name=\"hr_year-ActDes\")\n",

" df_total_act_per_inst.to_excel(writer, sheet_name=\"hr_year_inst-ActDes\")\n", " df_indiv_act.to_excel(writer, sheet_name=\"num_indiv-ActDes\")\n",

" df_ntp_act_inst.to_excel(writer, sheet_name=\"TNumPart_inst-ActDes\")\n", " df_nf_act_inst.to_excel(writer, sheet_name=\"NumFun_inst-ActDes\")\n",

" df_nnf_act_inst.to_excel(writer, sheet_name=\"NumNFun_inst-ActDes\")\n", " df_nnt_act_inst.to_excel(writer, sheet_name=\"NumNTrain_inst-ActDes\")\n", "\n",

"with pd.ExcelWriter(excel_prof) as writer:\n", " # ProfSkill related\n",

" df_id_prof.to_excel(writer, sheet_name=\"count-ID-ProfSkill\")\n", " df_total_prof.to_excel(writer, sheet_name=\"hr_year-ProfSkill\")\n",

" df_total_prof_per_inst.to_excel(writer, sheet_name=\"hr_year_inst-ProfSkill\")\n", " df_indiv_prof.to_excel(writer, sheet_name=\"num_indiv-ProfSkill\")\n",

" df_ntp_prof_inst.to_excel(writer, sheet_name=\"TNumPart_inst-ProfSkill\")\n", " df_nf_prof_inst.to_excel(writer, sheet_name=\"NumFun_inst-ProfSkill\")\n",

" df_nnf_prof_inst.to_excel(writer, sheet_name=\"NumNFun_inst-ProfSkill\")\n", " df_nnt_prof_inst.to_excel(writer, sheet_name=\"NumNTrain_inst-ProfSkill\")\n", "\n",

"with pd.ExcelWriter(excel_stk) as writer:\n", " # StkSrvd related\n",

" df_id_prof_stk.to_excel(writer, sheet_name=\"count-ID-StkSrvd\")\n", " df_total_stk.to_excel(writer, sheet_name=\"hr_year-StkSrvd\")\n",

" df_total_stk_per_inst.to_excel(writer, sheet_name=\"hr_year_inst-StkSrvd\")\n", " df_indiv_stk.to_excel(writer, sheet_name=\"num_indiv-StkSrvd\")\n",

" df_ntp_stk_inst.to_excel(writer, sheet_name=\"TNumPart_inst-StkSrvd\")\n", " df_nf_stk_inst.to_excel(writer, sheet_name=\"NumFun_inst-StkSrvd\")\n",

" df_nnf_stk_inst.to_excel(writer, sheet_name=\"NumNFun_inst-StkSrvd\")\n", " df_nnt_stk_inst.to_excel(writer, sheet_name=\"NumNTrain_inst-StkSrvd\")\n", "\n",

"with pd.ExcelWriter(excel_pair) as writer:\n", " # Pairwise\n",

" df_act_prof.to_excel(writer, sheet_name=\"count-ActDes-ProfSkill\")\n", " df_act_stk.to_excel(writer, sheet_name=\"count-ActDes-StkSrvd\")\n",

" df_prof_stk.to_excel(writer, sheet_name=\"count-ProfSkill-StkSrvd\")"

]

},

{

"cell_type": "markdown", "metadata": {},

"source": [

"## Testing "

]

},

{

"cell_type": "markdown", "metadata": {},

"source": [

"### Number of institution-activity"

]

},

{

"cell_type": "code", "execution_count": 40, "metadata": {},

"outputs": [], "source": [

"# Number of institutions offerring\n", "ser_act = data['ActDes'] # activities\n", "ser_iid = data['ID'] # institutional ID\n", "idx_act = cat_dict[\"ActDes\"].keys()\n", "\n",

"id_act_dict = {} # {institutional ID: {activity idx: count}}\n", "for idx, act_val in enumerate(ser_act):\n",

" iid = ser_iid[idx]\n", "\n",

" # Check if there are multiple activity codes\n", " if type(act_val) == str:\n",

" act_list = act_val.split(\",\")\n",

" act_list = [int(v) for v in act_list]\n", " else:\n",

" act_list = [act_val]\n", "\n",

" for act in act_list:\n",

" if iid not in id_act_dict:\n",

" id_act_dict[iid] = {act:1}\n",

" elif act not in id_act_dict[iid]:\n",

" id_act_dict[iid][act] = 1\n", " else:\n",

" id_act_dict[iid][act] += 1\n", "\n",

"# Fill in 0s\n",

"for act_idx in id_act_dict:\n",

" act_dict = id_act_dict[act_idx]\n", " for idx in idx_act:\n",

" if idx not in act_dict:\n", " act_dict[idx] = 0\n", "\n",

"# Genreate output\n",

"iid_list = list(id_act_dict.keys())\n", "iid_list.sort()\n",

"with open(work_dir / \"table_instID_actID.tsv\", \"w\") as f:\n", " f.write(\"InstID\\t\")\n",

" f.write(\"\\t\".join([str(i) for i in idx_act]))\n", " f.write(\"\\n\")\n",

" for iid in iid_list:\n", " f.write(str(iid))\n",

" idx_dict = id_act_dict[iid]\n", " for idx in idx_act:\n",

" f.write(f\"\\t{id_act_dict[iid][idx]}\")\n", " f.write(\"\\n\")"

]

},

{

"cell_type": "code", "execution_count": 41, "metadata": {}, "outputs": [

{

"ename": "NameError",

"evalue": "name 'col1' is not defined", "output_type": "error",

"traceback": [

"\u001b[0;31m \u001b[0m",

"\u001b[0;31mNameError\u001b[0m Traceback (most recent call last)", "\u001b[1;32m/home/shinhan/proj_onedrive/nrt/script_nrt_prof_dev_analysis.ipynb Cell 72\u001b[0m line

\u001b[0;36m2\n\u001b[1;32m <a href='vscode-notebook-cell://wsl%2Bubuntu-20.04/home/shinhan/proj_onedrive/nrt/script_nrt_prof_dev_analysis.ipynb#Y101sdnNjb2RlLXJlbW90ZQ%3D%3D? line=0'>1</a>\u001b[0m \u001b[39m# Genreate output\u001b[39;00m\n\u001b[0;32m > <a href='vscode-notebook-

cell://wsl%2Bubuntu-20.04/home/shinhan/proj_onedrive/nrt/script_nrt_prof_dev_analysis.ipynb#Y101sdnNjb2RlLXJlbW90ZQ%3D%3D? line=1'>2</a>\u001b[0m \u001b[39mwith\u001b[39;00m \u001b[39mopen\u001b[39m(work_dir

\u001b[39m/\u001b[39m

\u001b[39mf\u001b[39m\u001b[39m\"\u001b[39m\u001b[39mtable_\u001b[39m\u001b[39m{\u001b[39;00mcol1\u00 1b[39m}\u001b[39;00m\u001b[39m_\u001b[39m\u001b[39m{\u001b[39;00mcol2\u001b[39m}\u001b[39;00m\u001b[ 39m.tsv\u001b[39m\u001b[39m\"\u001b[39m,

\u001b[39m\"\u001b[39m\u001b[39mw\u001b[39m\u001b[39m\"\u001b[39m) \u001b[39mas\u001b[39;00m f:\n\u001b[1;32m <a href='vscode-notebook-cell://wsl%2Bubuntu-20.04/home/shinhan/proj_onedrive/nrt/script_nrt_prof_dev_analysis.ipynb#Y101sdnNjb2RlLXJlbW90ZQ%3D%3D? line=2'>3</a>\u001b[0m f\u001b[39m.\u001b[39mwrite(\u001b[39mf\u001b[39m\u001b[39m\"\u001b[39m\u001b[39m{\u001b[39;00mcol1\u0

01b[39m}\u001b[39;00m\u001b[39m\\\u001b[39m\u001b[39m{\u001b[39;00mcol2\u001b[39m}\u001b[39;00m\u001b [39m\\t\u001b[39;00m\u001b[39m\"\u001b[39m)\n\u001b[1;32m <a href='vscode-notebook-cell://wsl%2Bubuntu-20.04/home/shinhan/proj_onedrive/nrt/script_nrt_prof_dev_analysis.ipynb#Y101sdnNjb2RlLXJlbW90ZQ%3D%3D? line=3'>4</a>\u001b[0m f\u001b[39m.\u001b[39mwrite(\u001b[39m\"\u001b[39m\u001b[39m\\t\u001b[39;00m\u001b[39m\"\u001b[39m\u001 b[39m.\u001b[39mjoin([\u001b[39mstr\u001b[39m(i) \u001b[39mfor\u001b[39;00m i \u001b[39min\u001b[39;00m idx_ser2]))\n",

"\u001b[0;31mNameError\u001b[0m: name 'col1' is not defined"

]

}

],

"source": [

"# Genreate output\n",

"with open(work_dir / f\"table_{col1}_{col2}.tsv\", \"w\") as f:\n", " f.write(f\"{col1}\\{col2}\\t\")\n",

" f.write(\"\\t\".join([str(i) for i in idx_ser2]))\n", " f.write(\"\\n\")\n",

" for ser1_idx in idx_ser1:\n", " f.write(str(ser1_idx))\n",

" for ser2_idx in idx_ser2:\n",

" f.write(f\"\\t{dict_c1_c2[ser1_idx][ser2_idx]}\")\n", " f.write(\"\\n\")"

]

}

],

"metadata": { "kernelspec": {

"display_name": "sklearn", "language": "python", "name": "python3"

},

"language_info": { "codemirror_mode": { "name": "ipython", "version": 3

},

"file_extension": ".py", "mimetype": "text/x-python", "name": "python", "nbconvert_exporter": "python", "pygments_lexer": "ipython3", "version": "3.10.12"

}

},

"nbformat": 4,

"nbformat_minor": 2

}
