# Supplementary material for: Lessons learned from interdisciplinary US national science foundation research traineeship-supported graduate programs
Source: PLoS One. 2026 Feb 20;21(2):e0343307. doi: 10.1371/journal.pone.0343307 (PMC12923050; doi:10.1371/journal.pone.0343307)
Supplement: Attachment — Submitted filename: PONE Response.docx [file pone.0343307.s008.docx]

November 12, 2025

Joshua L. Rosenbloom
Academic Editor
PLOS ONE

Dear Dr. Rosenbloom,

Thank you for the opportunity to revise and resubmit our manuscript, *Lessons Learned from Interdisciplinary US National Science Foundation Research Traineeship-Supported Graduate Programs* (PONE-D-25-33375). Please find our response to reviewers below.

### Editor Comments

#### Funding

- Please add the following statement to our online submission: "The funders had no role in study design, data collection and analysis, decision to publish, or preparation of the manuscript."
- Please include both an updated Funding Statement and Competing Interests Statement in your cover letter. We will change the online submission form on your behalf.

We have included this information in our cover letter. And repeat here as well.

The funders had no role in study design, data collection and analysis, decision to publish, or preparation of the manuscript.

#### Competing Interests

- “The funder provided support in the form of salaries for authors [insert relevant initials], but did not have any additional role in the study design, data collection and analysis, decision to publish, or preparation of the manuscript. The specific roles of these authors are articulated in the ‘author contributions’ section.”
- please confirm that this commercial affiliation does not alter your adherence to all PLOS ONE policies on sharing data and materials by including the following statement: "This does not alter our adherence to PLOS ONE policies on sharing data and materials.”

We have included this information in our cover letter and repeat it here as well.The funder provided support in the form of salaries for authors [KM, SHS], but did not have any additional role in the study design, data collection and analysis, decision to publish, or preparation of the manuscript. The specific roles of these authors are articulated in the ‘author contributions’ section. This does not alter our adherence to PLOS ONE policies on sharing data and materials

#### Data

- please address the following prompts:

a) If there are ethical or legal restrictions on sharing a de-identified data set, please explain them in detail (e.g., data contain potentially identifying or sensitive patient information, data are owned by a third-party organization, etc.) and who has imposed them (e.g., a Research Ethics Committee or Institutional Review Board, etc.). Please also provide contact information for a data access committee, ethics committee, or other institutional body to which data requests may be sent.

b) If there are no restrictions, please upload the minimal anonymized data set necessary to replicate your study findings to a stable, public repository and provide us with the relevant URLs, DOIs, or accession numbers.

- include your tables as part of your main manuscript and remove the individual files. Please note that supplementary tables (should remain/ be uploaded) as separate "supporting information" files.
- captions for your Supporting Information files at the end of your manuscript, and update any in-text citations to match accordingly. Please see our Supporting Information guidelines for more information: <http://journals.plos.org/plosone/s/supporting-information>.

We have included a supplemental table file (S6) that has the data used in our analysis so that others can repeat the work, as appropriate. We have also included the tables in the main body of the text, removed the tables files, and added the supplementary tables as supporting information files with the captions at the end of the manuscript.

Reviewers' Comments

*Data*
R1 -- Request more data from the participating NRTs to enrich their analysis (e.g., job market outcomes, academic achievements, mental health).

We have collected additional information about each project from existing available information (e.g., project websites) that participated in this research and included project key words, geographical locations, R1/R2 data, and alignment to the NSF big ideas which provides more information than previously provided, without identifying the participating projects. Since the annual reports are the property of the individual project and the NSF, we did not have access to them, nor should we have as there is confidential and sensitive information included in these documents. Thus, we only solicited the required subset of Tables that the NSF requested from each project as part of their annual review submission. Please note, the NRT Program has a unique reporting requirement not typical of most NSF projects (see <https://nrtprogram.atlassian.net/wiki/spaces/NRTS/overview?homepageId=33164> ). Unfortunately, although a good idea, the reviewer’s request for more data is not possible as even if it were, many of the projects are expired and do not have existing presence (e.g., websites or personnel).

R2 -- p. 4, line 54: How many total programs did you solicit?

100 programs were included on the email listserve when we solicited the annual report tables from the projects. We have added this detail to the paper.

R2 -- The main problem is that the primary data source is NSF annual reports, which according to the Materials and Methods section, do not contain information on the **challenges faced** (R2 suggests possibly dropping).

We did not collect annual reports from the projects. We only collected Tables that were a required component of the annual reports from projects that were willing to provide them for this project. As such, we do not have information on challenges faced by projects, other than those projects that the authorship team represents. We have clarified this in the paper and expanded on the projects from the authorship team that provided the challenges faced. We have also expanded the information provided about all the projects Table 1 and Supplemental Table 2.

R1 -- does not inform the reader on how many programs did not agree to provide data. There **may be a selection bias** issue if the programs that accepted the invitation to share data differ systematically from those that did not.
Good point. We have added the following to the paper. Out of 100 programs we only got responses from the listed programs. However, we can not be sure it was because the others did not agree. Some of them did not provide the tables within our timeline of data collection. We asked 3x over 6 weeks (20% response rate). Listserve requests went to coordinators. Some projects just started and did not have the requested Tables since they had not submitted an annual report. Others may not have completed/submitted the tables at all in their submitted reports. The total programs that may have been able to respond was likely closer to 80% (25% response rate). We either received data or did not.

*Analysis*

R1 -- Statistical analysis is not rigorous. The authors **must complement the current frequency table and figures with more sophisticated tools, such as cluster analysis, principal component analysis, multilevel hierarchical models, and/or regression methods**. Cluster analysis would be helpful to identify patterns within specific programs. Principal component analysis could be used to reduce dimensionality (i.e., number of activities).

The goal of the frequency table and figures are to provide the readers with a holistic view of the dataset. Given the limited number of samples and variables, we respectfully disagree with the reviewer and feel that providing the data in tabular and figure formats would provide the readers the maximum amount of information. In addition, questions we ask drive how we conduct the analysis and generate visuals to address them which do not require the suggested analyses.

Taking Table 2 as an example, we address the question of which categories of stakeholders are involved the most among the programs we have data for. Without the additional analysis as suggested, Table 2 has already gotten the point across.

As for Figure 1, we want to answer the question what activity/skill combinations the analyzed programs tend to focus on. It’s a 11x14 matrix excluding the marginal distributions. Without any clustering, the heatmap clearly conveyed the information as intended and showed the major trends and correlated activities without clustering. The reviewer also suggested PCA in Figure 1. While PCA can be beneficial when the number of variables is large, there are only 10 activities. Besides, once the activities are reduced to fewer components, the principal components are combinations of activities that will not be as interpretable as the activities themselves.

R1-- Analysis section needs to describe the data-generating process better; it is **unclear how the authors generated the data used in the analysis**.

R1 – How the analysis section is written does not help the reader. I suggest the creation of a table to improve readability.

The details on how the analysis was done to generate these tables for the study is detailed in Supplement Table 4 with the script (Supplement Table 5). The anonymised data from Supplement Table 6 was used for the analysis. We have expanded our explanation and refer to the supplemental files to guide the reader and future analyses others may want to conduct.

R1 -- Color-coding of Figure 1 is not reader-friendly; better color scheme and/or a legend would assist the reader.

We regret that the color scheme was not considered reader-friendly. Our goal is to use the shade of red to indicate increasing numbers of activity/skill combinations. To allow comparison of the activities that tend to be used for providing the skill sets, on the right a %programs designed activities column is included with increasing shades of cyan to indicate increasing percentage. Similarly, shade of purple is used to indicate the increasing % of programs focused on skills. The colors were chosen to maximize the three aspects of the figure we intend to emphasize and to avoid color combinations that are not color-blind-friendly.

R2 -- There is no coding related to challenges or solutions.

We did not collect annual reports from the projects. We only collected Tables that were a required component of the annual reports from projects that were willing to provide them for this project. As such, we do not have information on challenges faced by projects, other than those projects that the authorship team represents. We have clarified this in the paper and expanded on the projects from the authorship team that provide examples of challenges faced. We have not coded this information since it represents our own experiences.

R2 -- p. 5, line 20: 42% is not most

We have changed “most” to “many”

R2 -- p. 6, Table 1: check your numbers here. An average of 123 "public stakeholders" is really a lot.

For more clarity, the reviewer should keep in mind that the number of individuals-stakeholder_type combined is not the raw count of individuals involved. For example, for an institution with three activities:

| **Row** | **StkSrvd** | **TNumPart** | **NumFun** | **NumNFun** | **NumNTrain** |
| --- | --- | --- | --- | --- | --- |
| 1 | 1,2 | 13 | 6 | 5 | 2 |
| 2 | 1,2 | 56 | 11 | 3 | 42 |
| 3 | 1 | 13 | 10 | 2 | 1 |

Then for stakeholders served (StkSrvd) with code of 1,the total number of participant-stakeholder_type is for the three activities (Rows): 13 + 56 + 13 (TNumPart). This analysis was conducted using the python code from the data in Supplement Table 6.

R2 -- p.6, line 46: **are you counting classes here?** That's where most of that technical training will occur, so including it only here seems a bit disingenuous

In the reported table, we coded for technical training based on skills listed by the programs. The programs could choose to report on required program course hours or not, but we only counted what they included. So, if they included course hours for the program skills, they would usually also report them for the disciplinary or traditional training hours as well.Thus, it was an “apples to apples” comparison.

*Discussion*
R1 -- no mention of artificial intelligence (AI) and how it shapes NRTs and STEM graduate education. The authors need to include AI and discuss its implications.

It has just been recently that NRT projects have had more of an emphasis of AI (e.g., almost half of the 2025 awards had such a focus). The data for this project were collected on projects in the reporting years of 2021-2023, where AI was not yet a major focus of NRT projects. However, we agree that AI is now a very important aspect of graduate training programs and have included this sentiment in our discussion.

R2 -- The results present no data related to challenges and solutions.

We did not collect annual reports from the projects. We only collected Tables that were a required component of the annual reports from projects that were willing to provide them for this project. As such, we do not have information on challenges faced by projects, other than those projects that the authorship team represents. We have clarified this in the paper and expanded on the projects from the authorship team and then provide examples of challenges faced based on our experiences. We agree that it is more interesting to include these challenges in the paper, rather than simply reporting on the tables collected from the annual reports. We unfortunately have no way to go back to the projects that provided the tables, as many of them no longer exist due to expiration of funding.

R2 -- Though there is information from three programs included in the discussion and conclusion, the reader has no way to know how that information was obtained and how it was analyzed. It's not useful as is. **One option would be to drop the claim of discussing challenges and solutions** and simply present your analysis of the annual reports, but I don't think this is much of a contribution. **The more interesting and effective approach would be to really expand the analysis of the three programs mentioned.** That means we need to know about them from the beginning, your **data collection needs to be discussed in the methods section**, we need to see **data reported in the results**, and then you can discuss them in the discussion - **they should also be put into conversation with the annual report data in order to really build an interesting argument and provide useful knowledge**.

We did not collect annual reports from the projects. We only collected Tables that were a NSF required component of the annual reports from projects that were willing to provide them for this project. As such, we do not have information on challenges faced by projects, other than those projects that the authorship team represents. We have clarified this in the paper and expanded on the projects from the authorship team and then provide examples of challenges faced based on our experiences. We agree that it is more interesting to include these challenges in the paper, rather than simply reporting on the tables collected from the annual reports. We unfortunately have no way to go back to the projects that provided the tables, as many of them no longer exist due to expiration of funding.

R2 -- p.7, lines 09-13. You state: "Findings from our analysis of 20 NRT programs suggest that it is feasible to move beyond traditional disciplinary-based content and to shape long-lasting change in graduate education to meet the growing need for scholars who are prepared to **join the professional workforce and to improve our understanding of complex socio-ecological problems**." But it's **not clear to me that you have presented evidence that shows that it is feasible** and it certainly doesn't show anything about long-lasting change in graduate education.

Table 1, which we have added to the paper to address this comment along with others, summarizes the three projects that the authorship team represent, we have added information about how each project has been institutionalized at their current university in Table 1. Institutionalization is a major component of NRT projects. Hence, it is important to address here, even if the data from annual reports were not collected in this project.

R2 -- p. 7, line 90: where are you getting your evidence for the claim that interdisciplinary programs lack faculty and institutional support? I don't have data here, but my experience has been that faculty *love* these programs, it's just the admin that is difficult.

We have clarified that the paper includes information from the three projects represented by the authorship team, included Table 1 describing each in more detail, and utilized these projects as case examples of challenges faced based on our experiences. One such challenge has included institutional support (or as you state administrator) for interdisciplinary programs which we have expanded on in the discussion section.

R2 – Misc, comments
- p. 3, line 33: How are you defining "successful" NRT programs
- p. 5, line 01: "one per year" ... one what? I'm really confused here.
We have corrected both of these editorial items in the paper. Thank you for catching them.
