# Supplementary material for: Lessons learned from interdisciplinary US national science foundation research traineeship-supported graduate programs
Source: PLoS One. 2026 Feb 20;21(2):e0343307. doi: 10.1371/journal.pone.0343307 (PMC12923050; doi:10.1371/journal.pone.0343307)
Supplement: Attachment — Submitted filename: Responses to Editor for 2nd revision.docx [file pone.0343307.s009.docx]

1. You state your goal as investigating how NRT programs can serve as a "model for innovative graduate education and interdisciplinary training..." (p. 11). It would be helpful for readers if you make more explicit in the introduction and discussion what advice you have in that regard. What elements of NRT programs (at least the three with which you are involved) are most effective? Is there any insight about the relative cost of these elements? In other words, I would like to see you draw clearer and more actionable conclusions, or else to acknowledge the difficulty of doing so given the limited information available. In the latter case, please reflect on what information is needed to better assess NRT programs.
   a. We have added a new section titled “Challenges: Observed at Three NRT Projects” to address this point in the discussion section. We have addressed costs in the “Program Scaling and Institutionalization” section of the discussion.
2. I recognize information is limited, but are there any comparative insights to be drawn across the three programs you represent?
   a. We have added significantly to the paper to address this concern. We have added a section titled “Three NRT Projects in Detail” that describes the three programs and highlights what they have in common and what is different, providing more insight into each program's characteristics. We have also added two new areas to Table 1 – “Outreach and internships” and “Institutional elements” to show more details and breadth about each of the three projects. Additionally, in the sections that follow, which describe each of our projects, we have added an “institutionalization” and a “challenges” section for each project. This has allowed us to expand the discussion section around barriers and solutions.
3. You are largely silent about the design and requirements of the NRT program. Do you believe that there are changes NSF can make that would lower the cost of effective programs or making them easier to implement.
   a. In the discussion, we have added a section “Solutions: Funder and University Engagement” which includes a discussion about Program Sustainability and Program Scaling which addresses some activities NSF could conduct.
4. The structure of the article makes it very hard to parse. Information needed to interpret Figure 1, for example, is buried in methods and materials at the end of the article. I suggest addressing these questions at the outset and contextualizing the objects of study by explicitly discussing the programs you contacted, the programs that responded, and the three that you represent.
   a. We have moved the information about Figure 1 in the main text, where now the 20 programs are described BEFORE the three training programs so that the Figure is addressed sooner in the paper.
   b. 2nd part - Comment is confusing. Specific institutional program details we cannot address because we are not allowed to share more identifiable information, as respondents were informed that they would not be identified in the analysis.
   c. We have added more information about the three main authors’ projects, however, to help address this point.
5. At a minimum the use of different colors in Figure 1 needs to be explained. Why are some cells red, and some blue? What are the units in which cell entries are measured?

We agree that further explanation would be helpful. We have added the following to the figure caption.

*Red: shade of red indicating lower (lighter) to higher (darker) number of instances for a particular activity/skill combination.*

*Purple: shade of purple indicating a lower to higher percentage of programs focused on a particular skill set.*

*Blue: shade of blue indicating a lower to higher percentage of programs conducting a particular activity*
