# Supplementary material for: Lessons learned from interdisciplinary US national science foundation research traineeship-supported graduate programs
Source: PLoS One. 2026 Feb 20;21(2):e0343307. doi: 10.1371/journal.pone.0343307 (PMC12923050; doi:10.1371/journal.pone.0343307)
Supplement: Peer review history

# Peer review: Decision Letter 0

**DOI:** 10.1371/journal.pone.0343307.r001

**Authors:** Joshua L Rosenbloom

28 Aug 2025

Dear Dr. McNeal,

Thank you for submitting your manuscript to PLOS ONE. After careful consideration, we feel that it has merit but does not fully meet PLOS ONE’s publication criteria as it currently stands. Therefore, we invite you to submit a revised version of the manuscript that addresses the points raised during the review process.

plosone@plos.org . When you're ready to submit your revision, log on to https://www.editorialmanager.com/pone/ and select the 'Submissions Needing Revision' folder to locate your manuscript file.

- A rebuttal letter that responds to each point raised by the academic editor and reviewer(s). You should upload this letter as a separate file labeled 'Response to Reviewers'.
- A marked-up copy of your manuscript that highlights changes made to the original version. You should upload this as a separate file labeled 'Revised Manuscript with Track Changes'.
- An unmarked version of your revised paper without tracked changes. You should upload this as a separate file labeled 'Manuscript'.

If you would like to make changes to your financial disclosure, please include your updated statement in your cover letter. Guidelines for resubmitting your figure files are available below the reviewer comments at the end of this letter.

If applicable, we recommend that you deposit your laboratory protocols in protocols.io to enhance the reproducibility of your results. Protocols.io assigns your protocol its own identifier (DOI) so that it can be cited independently in the future. For instructions see: https://journals.plos.org/plosone/s/submission-guidelines#loc-laboratory-protocols . Additionally, PLOS ONE offers an option for publishing peer-reviewed Lab Protocol articles, which describe protocols hosted on protocols.io. Read more information on sharing protocols at https://plos.org/protocols?utm_medium=editorial-email&utm_source=authorletters&utm_campaign=protocols .

We look forward to receiving your revised manuscript.

Kind regards,

Joshua L Rosenbloom

Academic Editor

PLOS ONE

Journal Requirements:

When submitting your revision, we need you to address these additional requirements.

1.Please ensure that your manuscript meets PLOS ONE's style requirements, including those for file naming. The PLOS ONE style templates can be found at

https://journals.plos.org/plosone/s/file?id=wjVg/PLOSOne_formatting_sample_main_body.pdf and

https://journals.plos.org/plosone/s/file?id=ba62/PLOSOne_formatting_sample_title_authors_affiliations.pdf

2. Thank you for stating the following financial disclosure:

“This work is supported by the National Science Foundation Research Traineeship

(NRT) with projects funded at Auburn University (Climate Resilience NRT, DGE1922687; PIs: McNeal and Mitra), Michigan State University (IMPACTS NRT, DGE1828149; PI: Shiu) and at Boston University (URBAN NRT, DGE-1735087, PI:

Templer).”

Please state what role the funders took in the study.  If the funders had no role, please state: "The funders had no role in study design, data collection and analysis, decision to publish, or preparation of the manuscript."

If this statement is not correct you must amend it as needed.

Please include this amended Role of Funder statement in your cover letter; we will change the online submission form on your behalf.

3. Thank you for stating the following in the Competing Interests section:

“While one of the authors is a current program director and another recently completed their program officer duties at NSF, neither of them were in these roles during the data collection and analysis. All other authors declare they have no competing interests.”

We note that one or more of the authors are employed by a commercial company: NSF

1. Please provide an amended Funding Statement declaring this commercial affiliation, as well as a statement regarding the Role of Funders in your study. If the funding organization did not play a role in the study design, data collection and analysis, decision to publish, or preparation of the manuscript and only provided financial support in the form of authors' salaries and/or research materials, please review your statements relating to the author contributions, and ensure you have specifically and accurately indicated the role(s) that these authors had in your study. You can update author roles in the Author Contributions section of the online submission form.

Please also include the following statement within your amended Funding Statement.

“The funder provided support in the form of salaries for authors [insert relevant initials], but did not have any additional role in the study design, data collection and analysis, decision to publish, or preparation of the manuscript. The specific roles of these authors are articulated in the ‘author contributions’ section.”

If your commercial affiliation did play a role in your study, please state and explain this role within your updated Funding Statement.

2. Please also provide an updated Competing Interests Statement declaring this commercial affiliation along with any other relevant declarations relating to employment, consultancy, patents, products in development, or marketed products, etc.

Within your Competing Interests Statement, please confirm that this commercial affiliation does not alter your adherence to all PLOS ONE policies on sharing data and materials by including the following statement: "This does not alter our adherence to  PLOS ONE policies on sharing data and materials.” (as detailed online in our guide for authors http://journals.plos.org/plosone/s/competing-interests) . If this adherence statement is not accurate and  there are restrictions on sharing of data and/or materials, please state these. Please note that we cannot proceed with consideration of your article until this information has been declared.

Please include both an updated Funding Statement and Competing Interests Statement in your cover letter. We will change the online submission form on your behalf.

4. We note that you have indicated that there are restrictions to data sharing for this study. PLOS only allows data to be available upon request if there are legal or ethical restrictions on sharing data publicly. For more information on unacceptable data access restrictions, please see http://journals.plos.org/plosone/s/data-availability#loc-unacceptable-data-access-restrictions.

Before we proceed with your manuscript, please address the following prompts:

a) If there are ethical or legal restrictions on sharing a de-identified data set, please explain them in detail (e.g., data contain potentially identifying or sensitive patient information, data are owned by a third-party organization, etc.) and who has imposed them (e.g., a Research Ethics Committee or Institutional Review Board, etc.). Please also provide contact information for a data access committee, ethics committee, or other institutional body to which data requests may be sent.

b) If there are no restrictions, please upload the minimal anonymized data set necessary to replicate your study findings to a stable, public repository and provide us with the relevant URLs, DOIs, or accession numbers. For a list of recommended repositories, please see

https://journals.plos.org/plosone/s/recommended-repositories. You also have the option of uploading the data as Supporting Information files, but we would recommend depositing data directly to a data repository if possible.

We will update your Data Availability statement on your behalf to reflect the information you provide.

5. Please include your tables as part of your main manuscript and remove the individual files. Please note that supplementary tables (should remain/ be uploaded) as separate "supporting information" files.

6. Please include captions for your Supporting Information files at the end of your manuscript, and update any in-text citations to match accordingly. Please see our Supporting Information guidelines for more information: http://journals.plos.org/plosone/s/supporting-information.

7. If the reviewer comments include a recommendation to cite specific previously published works, please review and evaluate these publications to determine whether they are relevant and should be cited. There is no requirement to cite these works unless the editor has indicated otherwise.

Additional Editor Comments:

You have two reviewers who are very sympathetic to the questions that your submission seeks to address.  They share your view that the topic is important.  Their feedback, included below, indicates that the current version falls far short of PLOS ONE criteria.  As they indicate the data you are considering may not be sufficient to answer the questions that you pose, and the statistical analysis is not appropriately performed.  Both reviewers provide extensive and explicit suggestions about how you might improve your analysis and I encourage you to consider and respond to these suggestions should you wish to submit a revised article.

[Note: HTML markup is below. Please do not edit.]

Reviewers' comments:

Reviewer's Responses to Questions

**Comments to the Author**

1. Is the manuscript technically sound, and do the data support the conclusions?

Reviewer #1: Partly

Reviewer #2: No

**********

2. Has the statistical analysis been performed appropriately and rigorously?

Reviewer #1: No

Reviewer #2: N/A

**********

3. Have the authors made all data underlying the findings in their manuscript fully available?

The PLOS Data policy

Reviewer #1: No

Reviewer #2: No

**********

4. Is the manuscript presented in an intelligible fashion and written in standard English?

Reviewer #1: Yes

Reviewer #2: Yes

**********

Reviewer #1: Referee report for PONE-D-25-33375 entitled "Lessons Learned from Interdisciplinary US National Science Foundation Research Traineeship-Supported Graduate Programs"

This paper investigates the skills provided by NSF NRT programs to trainees and how these skills are integrated with their home department education. It also assesses the targeted stakeholders and discusses implications and suggestions for successful interdisciplinarity and STEM graduate education in general. Combining data from 20 NRT Program Annual reports, the authors find that communication, job readiness, and team science are the activities with the most time allotted. Performing a multi-period coding task, the authors match the data from the various NRT programs and generate a table demonstrating the number of stakeholders and their attended activities, and two figures displaying the activities for training different professional skills and the time devoted to developing those skills. They suggest a need for change in graduate education to meet the demand for scholars better prepared to tackle complex socio-ecological problems.

The paper seeks to answer important research questions and provide insights into the NRT programming and STEM graduate education in general. While the introduction of the paper is well-written, several core aspects—particularly the analysis and results sections—need to be more clearly articulated and better developed. Please see my comments below.

Major Comments

Comment #1

While your contribution in examining the activities and skills developed via NRT programs is commendable, the statistical analysis is not rigorous. The authors must complement the current frequency table and figures with more sophisticated tools, such as cluster analysis, principal component analysis, multilevel hierarchical models, and/or regression methods. Cluster analysis would be helpful to identify patterns within specific programs. Principal component analysis could be used to reduce dimensionality (i.e., number of activities).

Comment #2

During an NRT program (24 months), participants fill out a series of questionnaires and surveys. I suggest the authors request more data from the participating NRTs to enrich their analysis. Some of the data could include job market outcomes, academic achievements, mental health, etc.

Comment #3

While the coding paragraph in the manuscript is well-written and underscores the difficulty of matching the results of the various reports, the analysis section needs to describe the data-generating process better. Even after exploring carefully the supplementary materials, it is unclear how the authors generated the data used in the analysis.

Comment #4

While the study utilizes data from 20 different NRT programs in 12 states, it does not inform the reader on how many programs did not agree to provide data. In line 152, on page 4 of 11 of the main manuscript, it is mentioned that data was solicited from all currently (2021-2022) funded programs. There may be a selection bias issue if the programs that accepted the invitation to share data differ systematically from those that did not.

Comment #5

The discussion section is quite extensive. While the detailed context you provide is appreciated, there is no mention of artificial intelligence (AI) and how it shapes NRTs and STEM graduate education. A large number of the currently funded programs incorporate elements of AI in their programming. The authors need to include AI and discuss its implications.

Minor Comments

Comment #6

The color-coding of Figure 1 is not reader-friendly. A better color scheme and/or a legend would assist the reader substantially.

Comment #7

How the analysis section is written does not help the reader. I suggest the creation of a table to improve readability.

Reviewer #2: This paper aims to assess approaches to providing interdisciplinary training in the context of NRT programs, show how these programs can serve as models for this type of training, and discusses challenges and solutions for interdisciplinary training. These are laudable and important goals, however the paper falls short of meeting them. The main problem is that the primary data source is NSF annual reports, which according to the Materials and Methods section, do not contain information on the challenges faced. Furthermore, there is no coding related to challenges or solutions. The results present no data related to challenges and solutions. Though there is information from three programs included in the discussion and conclusion, the reader has no way to know how that information was obtained and how it was analyzed. It's not useful as is.

One option would be to drop the claim of discussing challenges and solutions and simply present your analysis of the annual reports, but I don't think this is much of a contribution. The more interesting and effective approach would be to really expand the analysis of the three programs mentioned. That means we need to know about them from the beginning, your data collection needs to be discussed in the methods section, we need to see data reported in the results, and then you can discuss them in the discussion - they should also be put into conversation with the annual report data in order to really build an interesting argument and provide useful knowledge.

A few other specific comments:

- p. 3, line 33: How are you defining "successful" NRT programs

- p. 4, line 54: How many total programs did you solicit?

- p. 5, line 01: "one per year" ... one what? I'm really confused here.

- p. 5, line 20: 42% is not most

- p.6, line 46: are you counting classes here? That's where most of that technical training will occur, so including it only here seems a bit disingenuous

- p. 6, Table 1: check your numbers here. An average of 123 "public stakeholders" is really a lot.

- p. 7, line 90: where are you getting your evidence for the claim that interdisciplinary programs lack faculty and institutional support? I don't have data here, but my experience has bee that faculty *love* these programs, it's just the admin that is difficult.

- p.7, lines 09-13. You state: "Findings from our analysis of 20 NRT programs suggest that it is feasible to move beyond traditional disciplinary-baed content and to shape long-lasting change in graduate education to meet the growing need for scholars who are prepared to join the professional workforce and to improve our understanding of complex socio-ecological problems." But it's not clear to me that you have presented evidence that shows that it is feasible and it certainly doesn't show anything about long-lasting change in graduate education.

**********

what does this mean? ). If published, this will include your full peer review and any attached files.

If you choose “no”, your identity will remain anonymous but your review may still be made public.

**Do you want your identity to be public for this peer review?** For information about this choice, including consent withdrawal, please see our Privacy Policy

Reviewer #1: No

Reviewer #2: No

**********

[NOTE: If reviewer comments were submitted as an attachment file, they will be attached to this email and accessible via the submission site. Please log into your account, locate the manuscript record, and check for the action link "View Attachments". If this link does not appear, there are no attachment files.]

While revising your submission, please upload your figure files to the Preflight Analysis and Conversion Engine (PACE) digital diagnostic tool, https://pacev2.apexcovantage.com/ . PACE helps ensure that figures meet PLOS requirements. To use PACE, you must first register as a user. Registration is free. Then, login and navigate to the UPLOAD tab, where you will find detailed instructions on how to use the tool. If you encounter any issues or have any questions when using PACE, please email PLOS at figures@plos.org . Please note that Supporting Information files do not need this step.

# Author comment: Author response to Decision Letter 1

**DOI:** 10.1371/journal.pone.0343307.r002

1 Dec 2025

Reviewers' Comments

Data

R1 -- Request more data from the participating NRTs to enrich their analysis (e.g., job market outcomes, academic achievements, mental health).

We have collected additional information about each project from existing available information (e.g., project websites) that participated in this research and included project key words, geographical locations, R1/R2 data, and alignment to the NSF big ideas which provides more information than previously provided, without identifying the participating projects. Since the annual reports are the property of the individual project and the NSF, we did not have access to them, nor should we have as there is confidential and sensitive information included in these documents. Thus, we only solicited the required subset of Tables that the NSF requested from each project as part of their annual review submission. Please note, the NRT Program has a unique reporting requirement not typical of most NSF projects (see https://nrtprogram.atlassian.net/wiki/spaces/NRTS/overview?homepageId=33164 ). Unfortunately, although a good idea, the reviewer’s request for more data is not possible as even if it were, many of the projects are expired and do not have existing presence (e.g., websites or personnel).

R2 -- p. 4, line 54: How many total programs did you solicit?

100 programs were included on the email listserve when we solicited the annual report tables from the projects. We have added this detail to the paper.

R2 -- The main problem is that the primary data source is NSF annual reports, which according to the Materials and Methods section, do not contain information on the challenges faced (R2 suggests possibly dropping).

We did not collect annual reports from the projects. We only collected Tables that were a required component of the annual reports from projects that were willing to provide them for this project. As such, we do not have information on challenges faced by projects, other than those projects that the authorship team represents. We have clarified this in the paper and expanded on the projects from the authorship team that provided the challenges faced. We have also expanded the information provided about all the projects Table 1 and Supplemental Table 2.

R1 -- does not inform the reader on how many programs did not agree to provide data. There may be a selection bias issue if the programs that accepted the invitation to share data differ systematically from those that did not.

Good point. We have added the following to the paper. Out of 100 programs we only got responses from the listed programs. However, we can not be sure it was because the others did not agree. Some of them did not provide the tables within our timeline of data collection. We asked 3x over 6 weeks (20% response rate). Listserve requests went to coordinators. Some projects just started and did not have the requested Tables since they had not submitted an annual report. Others may not have completed/submitted the tables at all in their submitted reports. The total programs that may have been able to respond was likely closer to 80% (25% response rate). We either received data or did not.

Analysis

R1 -- Statistical analysis is not rigorous. The authors must complement the current frequency table and figures with more sophisticated tools, such as cluster analysis, principal component analysis, multilevel hierarchical models, and/or regression methods. Cluster analysis would be helpful to identify patterns within specific programs. Principal component analysis could be used to reduce dimensionality (i.e., number of activities).

The goal of the frequency table and figures are to provide the readers with a holistic view of the dataset. Given the limited number of samples and variables, we respectfully disagree with the reviewer and feel that providing the data in tabular and figure formats would provide the readers the maximum amount of information. In addition, questions we ask drive how we conduct the analysis and generate visuals to address them which do not require the suggested analyses.

Taking Table 2 as an example, we address the question of which categories of stakeholders are involved the most among the programs we have data for. Without the additional analysis as suggested, Table 2 has already gotten the point across.

As for Figure 1, we want to answer the question what activity/skill combinations the analyzed programs tend to focus on. It’s a 11x14 matrix excluding the marginal distributions. Without any clustering, the heatmap clearly conveyed the information as intended and showed the major trends and correlated activities without clustering. The reviewer also suggested PCA in Figure 1. While PCA can be beneficial when the number of variables is large, there are only 10 activities. Besides, once the activities are reduced to fewer components, the principal components are combinations of activities that will not be as interpretable as the activities themselves.

R1-- Analysis section needs to describe the data-generating process better; it is unclear how the authors generated the data used in the analysis.

R1 – How the analysis section is written does not help the reader. I suggest the creation of a table to improve readability.

The details on how the analysis was done to generate these tables for the study is detailed in Supplement Table 4 with the script (Supplement Table 5). The anonymised data from Supplement Table 6 was used for the analysis. We have expanded our explanation and refer to the supplemental files to guide the reader and future analyses others may want to conduct.

R1 -- Color-coding of Figure 1 is not reader-friendly; better color scheme and/or a legend would assist the reader.

We regret that the color scheme was not considered reader-friendly. Our goal is to use the shade of red to indicate increasing numbers of activity/skill combinations. To allow comparison of the activities that tend to be used for providing the skill sets, on the right a %programs designed activities column is included with increasing shades of cyan to indicate increasing percentage. Similarly, shade of purple is used to indicate the increasing % of programs focused on skills. The colors were chosen to maximize the three aspects of the figure we intend to emphasize and to avoid color combinations that are not color-blind-friendly.

R2 -- There is no coding related to challenges or solutions.

We did not collect annual reports from the projects. We only collected Tables that were a required component of the annual reports from projects that were willing to provide them for this project. As such, we do not have information on challenges faced by projects, other than those projects that the authorship team represents. We have clarified this in the paper and expanded on the projects from the authorship team that provide examples of challenges faced. We have not coded this information since it represents our own experiences.

R2 -- p. 5, line 20: 42% is not most

We have changed “most” to “many”

R2 -- p. 6, Table 1: check your numbers here. An average of 123 "public stakeholders" is really a lot.

For more clarity, the reviewer should keep in mind that the number of individuals-stakeholder_type combined is not the raw count of individuals involved. For example, for an institution with three activities:

Row

StkSrvd

TNumPart

NumFun

NumNFun

NumNTrain

1

1,2

13

6

5

2

2

1,2

56

11

3

42

3

1

13

10

2

1

Then for stakeholders served (StkSrvd) with code of 1,the total number of participant-stakeholder_type is for the three activities (Rows): 13 + 56 + 13 (TNumPart). This analysis was conducted using the python code from the data in Supplement Table 6.

R2 -- p.6, line 46: are you counting classes here? That's where most of that technical training will occur, so including it only here seems a bit disingenuous

In the reported table, we coded for technical training based on skills listed by the programs. The programs could choose to report on required program course hours or not, but we only counted what they included. So, if they included course hours for the program skills, they would usually also report them for the disciplinary or traditional training hours as well.Thus, it was an “apples to apples” comparison.

Discussion

R1 -- no mention of artificial intelligence (AI) and how it shapes NRTs and STEM graduate education. The authors need to include AI and discuss its implications.

It has just been recently that NRT projects have had more of an emphasis of AI (e.g., almost half of the 2025 awards had such a focus). The data for this project were collected on projects in the reporting years of 2021-2023, where AI was not yet a major focus of NRT projects. However, we agree that AI is now a very important aspect of graduate training programs and have included this sentiment in our discussion.

R2 -- The results present no data related to challenges and solutions.

We did not collect annual reports from the projects. We only collected Tables that were a required component of the annual reports from projects that were willing to provide them for this project. As such, we do not have information on challenges faced by projects, other than those projects that the authorship team represents. We have clarified this in the paper and expanded on the projects from the authorship team and then provide examples of challenges faced based on our experiences. We agree that it is more interesting to include these challenges in the paper, rather than simply reporting on the tables collected from the annual reports. We unfortunately have no way to go back to the projects that provided the tables, as many of them no longer exist due to expiration of funding.

R2 -- Though there is information from three programs included in the discussion and conclusion, the reader has no way to know how that information was obtained and how it was analyzed. It's not useful as is. One option would be to drop the claim of discussing challenges and solutions and simply present your analysis of the annual reports, but I don't think this is much of a contribution. The more interesting and effective approach would be to really expand the analysis of the three programs mentioned. That means we need to know about them from the beginning, your data collection needs to be discussed in the methods section, we need to see data reported in the results, and then you can discuss them in the discussion - they should also be put into conversation with the annual report data in order to really build an interesting argument and provide useful knowledge.

We did not collect annual reports from the projects. We only collected Tables that were a NSF required component of the annual reports from projects that were willing to provide them for this project. As such, we do not have information on challenges faced by projects, other than those projects that the authorship team represents. We have clarified this in the paper and expanded on the projects from the authorship team and then provide examples of challenges faced based on our experiences. We agree that it is more interesting to include these challenges in the paper, rather than simply reporting on the tables collected from the annual reports. We unfortunately have no way to go back to the projects that provided the tables, as many of them no longer exist due to expiration of funding.

R2 -- p.7, lines 09-13. You state: "Findings from our analysis of 20 NRT programs suggest that it is feasible to move beyond traditional disciplinary-based content and to shape long-lasting change in graduate education to meet the growing need for scholars who are prepared to join the professional workforce and to improve our understanding of complex socio-ecological problems." But it's not clear to me that you have presented evidence that shows that it is feasible and it certainly doesn't show anything about long-lasting change in graduate education.

Table 1, which we have added to the paper to address this comment along with others, summarizes the three projects that the authorship team represent, we have added information about how each project has been institutionalized at their current university in Table 1. Institutionalization is a major component of NRT projects. Hence, it is important to address here, even if the data from annual reports were not collected in this project.

R2 -- p. 7, line 90: where are you getting your evidence for the claim that interdisciplinary programs lack faculty and institutional support? I don't have data here, but my experience has been that faculty *love* these programs, it's just the admin that is difficult.

We have clarified that the paper includes information from the three projects represented by the authorship team, included Table 1 describing each in more detail, and utilized these projects as case examples of challenges faced based on our experiences. One such challenge has included institutional support (or as you state administrator) for interdisciplinary programs which we have expanded on in the discussion section.

R2 – Misc, comments

- p. 3, line 33: How are you defining "successful" NRT programs

- p. 5, line 01: "one per year" ... one what? I'm really confused here.

We have corrected both of these editorial items in the paper. Thank you for catching them.

Attachment Submitted filename: PONE Response.docx

# Peer review: Decision Letter 1

**DOI:** 10.1371/journal.pone.0343307.r003

**Authors:** Joshua L Rosenbloom

9 Dec 2025

Dear Dr. McNeal,

Thank you for submitting your manuscript to PLOS ONE. After careful consideration, we feel that it has merit but does not fully meet PLOS ONE’s publication criteria as it currently stands. Therefore, we invite you to submit a revised version of the manuscript that addresses the points raised during the review process.

==============================

1. You state your goal as investigating how NRT programs can serve as a "model for innovative graduate education and interdisciplinary training..." (p. 11).  It would be helpful for readers if you make more explicit in the introduction and discussion what advice you have in that regard.  What elements of NRT programs (at least the three with which you are involved) are most effective?  Is there any insight about the relative cost of these elements?  In other words, I would like to see you draw clearer and more actionable conclusions, or else to acknowledge the difficulty of doing so given the limited information available.  In the latter case, please reflect on what information is needed to better assess NRT programs.
2. I recognize information is limited, but are there any comparative insights to be drawn across the three programs you represent?
3. You are largely silent about the design and requirements of the NRT program.  Do you believe that there are changes NSF can make that would lower the cost of effective programs or making them easier to implement.
4. The structure of the article makes it very hard to parse.  Information needed to interpret Figure 1, for example, is buried in methods and materials at the end of the article.  I suggest addressing these questions at the outset, and contextualizing the objects of study by explicitly discussing the programs you contacted, the programs that responded, and the three that you represent.
5. At a minimum the use of different colors in Figure 1 needs to be explained.  Why are some cells red, and some blue?  What are the units in which cell entries are measured?

Please submit your revised manuscript by Jan 23 2026 11:59PM. If you will need more time than this to complete your revisions, please reply to this message or contact the journal office at plosone@plos.org . When you're ready to submit your revision, log on to https://www.editorialmanager.com/pone/ and select the 'Submissions Needing Revision' folder to locate your manuscript file.

- A rebuttal letter that responds to each point raised by the academic editor and reviewer(s). You should upload this letter as a separate file labeled 'Response to Reviewers'.
- A marked-up copy of your manuscript that highlights changes made to the original version. You should upload this as a separate file labeled 'Revised Manuscript with Track Changes'.
- An unmarked version of your revised paper without tracked changes. You should upload this as a separate file labeled 'Manuscript'.

If applicable, we recommend that you deposit your laboratory protocols in protocols.io to enhance the reproducibility of your results. Protocols.io assigns your protocol its own identifier (DOI) so that it can be cited independently in the future. For instructions see: https://journals.plos.org/plosone/s/submission-guidelines#loc-laboratory-protocols . Additionally, PLOS ONE offers an option for publishing peer-reviewed Lab Protocol articles, which describe protocols hosted on protocols.io. Read more information on sharing protocols at https://plos.org/protocols?utm_medium=editorial-email&utm_source=authorletters&utm_campaign=protocols .

We look forward to receiving your revised manuscript.

Kind regards,

Joshua L Rosenbloom

Academic Editor

PLOS One

Journal Requirements:

If the reviewer comments include a recommendation to cite specific previously published works, please review and evaluate these publications to determine whether they are relevant and should be cited. There is no requirement to cite these works unless the editor has indicated otherwise.

Please review your reference list to ensure that it is complete and correct. If you have cited papers that have been retracted, please include the rationale for doing so in the manuscript text, or remove these references and replace them with relevant current references. Any changes to the reference list should be mentioned in the rebuttal letter that accompanies your revised manuscript. If you need to cite a retracted article, indicate the article’s retracted status in the References list and also include a citation and full reference for the retraction notice.

Additional Editor Comments (if provided):

[Note: HTML markup is below. Please do not edit.]

Reviewers' comments:

Reviewer's Responses to Questions

**Comments to the Author**

Reviewer #1: All comments have been addressed

**********

2. Is the manuscript technically sound, and do the data support the conclusions?

Reviewer #1: Partly

**********

3. Has the statistical analysis been performed appropriately and rigorously?

Reviewer #1: N/A

**********

4. Have the authors made all data underlying the findings in their manuscript fully available?

The PLOS Data policy

Reviewer #1: No

**********

5. Is the manuscript presented in an intelligible fashion and written in standard English?

Reviewer #1: Yes

**********

Reviewer #1: (No Response)

**********

what does this mean? ). If published, this will include your full peer review and any attached files.

If you choose “no”, your identity will remain anonymous but your review may still be made public.

**Do you want your identity to be public for this peer review?** For information about this choice, including consent withdrawal, please see our Privacy Policy

Reviewer #1: No

**********

[NOTE: If reviewer comments were submitted as an attachment file, they will be attached to this email and accessible via the submission site. Please log into your account, locate the manuscript record, and check for the action link "View Attachments". If this link does not appear, there are no attachment files.]

To ensure your figures meet our technical requirements, please review our figure guidelines: https://journals.plos.org/plosone/s/figures

You may also use PLOS’s free figure tool, NAAS, to help you prepare publication quality figures: https://journals.plos.org/plosone/s/figures#loc-tools-for-figure-preparation.

NAAS will assess whether your figures meet our technical requirements by comparing each figure against our figure specifications.

# Author comment: Author response to Decision Letter 2

**DOI:** 10.1371/journal.pone.0343307.r004

30 Jan 2026

1a. We have added a new section titled “Challenges: Observed at Three NRT Projects” to address this point in the discussion section. We have addressed costs in the “Program Scaling and Institutionalization” section of the discussion.

2a. We have added significantly to the paper to address this concern. We have added a section titled “Three NRT Projects in Detail” that describes the three programs and highlights what they have in common and what is different, providing more insight into each program's characteristics. We have also added two new areas to Table 1 – “Outreach and internships” and “Institutional elements” to show more details and breadth about each of the three projects. Additionally, in the sections that follow, which describe each of our projects, we have added an “institutionalization” and a “challenges” section for each project. This has allowed us to expand the discussion section around barriers and solutions.

3a. In the discussion, we have added a section “Solutions: Funder and University Engagement” which includes a discussion about Program Sustainability and Program Scaling which addresses some activities NSF could conduct.

4a. We have moved the information about Figure 1 in the main text, where now the 20 programs are described BEFORE the three training programs so that the Figure is addressed sooner in the paper.

b. 2nd part - Comment is confusing. Specific institutional program details we cannot address because we are not allowed to share more identifiable information, as respondents were informed that they would not be identified in the analysis.

c. We have added more information about the three main authors’ projects, however, to help address this point.

5. We agree that further explanation would be helpful. We have added the following to the figure caption.

Red: shade of red indicating lower (lighter) to higher (darker) number of instances for a particular activity/skill combination.

Purple: shade of purple indicating a lower to higher percentage of programs focused on a particular skill set.

Blue: shade of blue indicating a lower to higher percentage of programs conducting a particular activity

Attachment Submitted filename: Responses to Editor for 2nd revision.docx

# Editor report: Decision Letter 2

**DOI:** 10.1371/journal.pone.0343307.r005

**Authors:** Joshua L Rosenbloom

5 Feb 2026

Lessons Learned from Interdisciplinary US National Science Foundation Research Traineeship-Supported Graduate Programs

PONE-D-25-33375R2

Dear Dr. McNeal,

We’re pleased to inform you that your manuscript has been judged scientifically suitable for publication and will be formally accepted for publication once it meets all outstanding technical requirements.

Thank you for the additional work that went into this final round of revision.  I think, and hope you agree, that the product is likely to be more impactful for this additional effort.

Within one week, you’ll receive an e-mail detailing the required amendments. When these have been addressed, you’ll receive a formal acceptance letter and your manuscript will be scheduled for publication.

An invoice will be generated when your article is formally accepted. Please note, if your institution has a publishing partnership with PLOS and your article meets the relevant criteria, all or part of your publication costs will be covered. Please make sure your user information is up-to-date by logging into Editorial Manager at Editorial Manager®  and clicking the ‘Update My Information' link at the top of the page. For questions related to billing, please contact billing support .

If your institution or institutions have a press office, please notify them about your upcoming paper to help maximize its impact. If they’ll be preparing press materials, please inform our press team as soon as possible -- no later than 48 hours after receiving the formal acceptance. Your manuscript will remain under strict press embargo until 2 pm Eastern Time on the date of publication. For more information, please contact onepress@plos.org.

Kind regards,

Joshua L Rosenbloom

Academic Editor

PLOS One

Additional Editor Comments (optional):

Reviewers' comments:

# Editor report: Acceptance letter

**DOI:** 10.1371/journal.pone.0343307.r006

**Authors:** Joshua L Rosenbloom

PONE-D-25-33375R2

PLOS One

Dear Dr. McNeal,

I'm pleased to inform you that your manuscript has been deemed suitable for publication in PLOS One. Congratulations! Your manuscript is now being handed over to our production team.

At this stage, our production department will prepare your paper for publication. This includes ensuring the following:

* All references, tables, and figures are properly cited

* All relevant supporting information is included in the manuscript submission,

* There are no issues that prevent the paper from being properly typeset

You will receive further instructions from the production team, including instructions on how to review your proof when it is ready. Please keep in mind that we are working through a large volume of accepted articles, so please give us a few days to review your paper and let you know the next and final steps.

Lastly, if your institution or institutions have a press office, please let them know about your upcoming paper now to help maximize its impact. If they'll be preparing press materials, please inform our press team within the next 48 hours. Your manuscript will remain under strict press embargo until 2 pm Eastern Time on the date of publication. For more information, please contact onepress@plos.org.

You will receive an invoice from PLOS for your publication fee after your manuscript has reached the completed accept phase. If you receive an email requesting payment before acceptance or for any other service, this may be a phishing scheme. Learn how to identify phishing emails and protect your accounts at https://explore.plos.org/phishing.

If we can help with anything else, please email us at customercare@plos.org.

Thank you for submitting your work to PLOS ONE and supporting open access.

Kind regards,

PLOS ONE Editorial Office Staff

on behalf of

Dr. Joshua L Rosenbloom

Academic Editor

PLOS One
